# Hematological Neoplasms with Eosinophilia

**DOI:** 10.3390/cancers16020337

**Published:** 2024-01-12

**Authors:** Rosario M. Morales-Camacho, Teresa Caballero-Velázquez, Juan José Borrero, Ricardo Bernal, Concepción Prats-Martín

**Affiliations:** 1 Department of Hematology, Virgen del Rocío University Hospital, Seville Biomedicine Institute (IBiS/CSIC), University of Seville, 41013 Seville, Spainricardobernal1@gmail.com (R.B.); 2 Department of Pathology, Virgen del Rocío University Hospital, 41013 Seville, Spain; jborreromt@yahoo.es

**Keywords:** eosinophilia, hematological neoplasm, myeloid/lymphoid neoplasm with eosinophilia, tyrosine kinase gene fusions, acute leukemia

## Abstract

**Simple Summary:**

The diagnostic assessment of eosinophilias is complex, requiring a multidisciplinary approach and often involving diagnostic challenges. This work aims for a better understanding of the cytomorphological features, immunophenotype, and biological activity of the eosinophil. Additionally, the concepts of peripheral and bone marrow eosinophilia and their potential causes are reviewed. Finally, the review focuses on the broad differential diagnosis of hematologic diseases that may underlie eosinophilia and how to diagnose them. Among the findings that should raise suspicion of hematologic diseases associated with eosinophilia are the presence of splenomegaly and/or lymphadenopathy or an abnormal blood count. In recent years, with advances in molecular techniques, new hematologic malignancies such as myeloid/lymphoid neoplasms with eosinophilia and tyrosine kinase gene fusions are being defined, where eosinophilia can serve as a guiding sign. In these cases, an accurate diagnosis allows for the use of targeted therapy with an improvement in the quality of life and survival of patients.

**Abstract:**

Eosinophils in peripheral blood account for 0.3–5% of leukocytes, which is equivalent to 0.05–0.5 × 10^9^/L. A count above 0.5 × 10^9^/L is considered to indicate eosinophilia, while a count equal to or above 1.5 × 10^9^/L is defined as hypereosinophilia. In bone marrow aspirate, eosinophilia is considered when eosinophils make up more than 6% of the total nuclear cells. In daily clinical practice, the most common causes of reactive eosinophilia are non-hematologic, whether they are non-neoplastic (allergic diseases, drugs, infections, or immunological diseases) or neoplastic (solid tumors). Eosinophilia that is associated with a hematological malignancy may be reactive or secondary to the production of eosinophilopoietic cytokines, and this is mainly seen in lymphoid neoplasms (Hodgkin lymphoma, mature T-cell neoplasms, lymphocytic variant of hypereosinophilic syndrome, and B-acute lymphoblastic leukemia/lymphoma). Eosinophilia that is associated with a hematological malignancy may also be neoplastic or primary, derived from the malignant clone, usually in myeloid neoplasms or with its origin in stem cells (myeloid/lymphoid neoplasms with eosinophilia and tyrosine kinase gene fusions, acute myeloid leukemia with core binding factor translocations, mastocytosis, myeloproliferative neoplasms, myelodysplastic/myeloproliferative neoplasms, and myelodysplastic neoplasms). There are no concrete data in standardized cytological and cytometric procedures that could predict whether eosinophilia is reactive or clonal. The verification is usually indirect, based on the categorization of the accompanying hematologic malignancy. This review focuses on the broad differential diagnosis of hematological malignancies with eosinophilia.

## 1. Introduction

### 1.1. The Eosinophil

Eosinophils are granular leukocytes originating in the bone marrow. The term “eosinophil” was coined by Paul Ehrlich in 1879 when he observed that the granules of these cells stained with acidic dyes, specifically with eosin Y (tetrabromofluorescein), producing a striking bright orange color [1]. However, it is highly likely that other researchers had observed this cell before Ehrlich [2].

Eosinophils should be considered tissue cells, with their presence in the blood being only circumstantial. They originate in the bone marrow from a pluripotent stem cell, which gives rise to a pluripotent myeloid progenitor cell, and this, in turn, gives rise to a specific or committed precursor in the genesis of the eosinophil granulocyte. Cytokines such as interleukin 3 (IL-3), interleukin 5 (IL-5) (the most specific to the eosinophil lineage), granulocyte-macrophage colony-stimulating factor (GM-CSF), or interleukin 33 (IL-33) act on this, regulating their maturation and differentiation [3,4]. After entering the bloodstream (hours), they migrate to various tissues. They are attracted in response to chemokines, primarily those of the eotaxin family (eotaxin-1/CCL11, eotaxin-2/CCL24, eotaxin-3/CCL26, and RANTES/CCL5), which activate the CCR3 receptor (C-C motif chemokine receptor 3) expressed on eosinophils. These chemokines are produced by fibroblasts, epithelial cells, endothelial cells, smooth muscle cells, T lymphocytes, and macrophages [5]. In the same way, complement C5a and lipid mediators have been linked as chemotactic factors [6]. Once in the tissues, eosinophils can survive for several weeks. They are preferentially present in the mucosa of the respiratory, digestive, and genitourinary tracts.

### 1.2. Eosinophil Biological Activity

Eosinophils process a single type of granulation, with heterogeneous content in their primordial stage (pre-eosinophil), which later condenses and crystallizes into what is referred to as specific granulation [7]. In terms of ultrastructure, these granules possess a central electrondense crystalline core surrounded by a matrix, and they are rich in cationic proteins that are highly toxic to parasites (helminths), as well as to bacteria, fungi, and viruses. The “major basic protein” is concentrated in the central crystalline core, while the matrix contains the “eosinophil-derived neurotoxin” or ribonuclease 2, the “cationic protein of the eosinophil” or ribonuclease 3, and the “eosinophil peroxidase” that generates potent oxidants. They also contain galectin-10, or “Charcot-Leyden protein,” which can be observed when it is crystallized in bone marrow aspirates and in tissues affected by eosinophilic infiltration [8]. In addition, mediators such as cytokines, chemokines, growth factors, lipid mediators, as well as multiple receptors for these, and enzymes are found. Thus, they play an immunomodulatory role in innate and adaptive immune responses, have anti-inflammatory and antitumor activity, and mediate the repair, remodeling, and maintenance of tissues and their homeostasis [9].

However, when eosinophils undergo massive and persistent activation, they can damage the tissues and organs in which they operate due to the toxicity of their content. This is what happens in hypereosinophilic syndromes or immediate hypersensitivity reactions (type I). A proinflammatory and prothrombotic state is generated and transforming growth factor-beta and interleukins are released, promoting the proliferation and activation of fibroblasts, leading to subsequent fibrosis at the expense of the parenchyma [9].

### 1.3. Normal and Pathological Eosinophil Morphology

**Figure 1:**
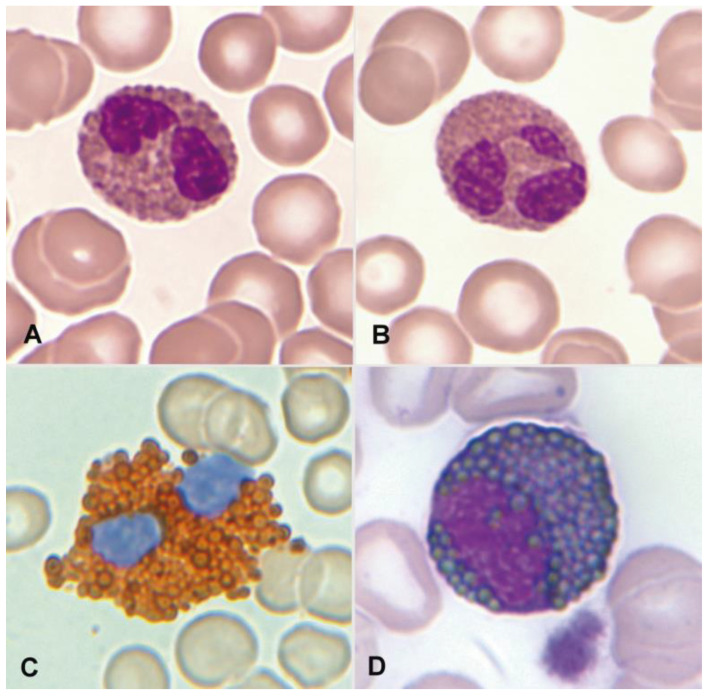
(**A**) Bilobed eosinophil. (**B**) Trilobed eosinophil. (**C**) Eosinophil with high myeloperoxidase content. (**D**) Eosinophil myelocyte which is Sudan Black B-positive.

In blood smears that have been fixed with methanol and stained with Romanowsky stains, the normal mature eosinophil reaches a size between 14 and 16 µm. The nucleus is bilobed, classically described as eyeglass-shaped, and sometimes trilobed. The cytoplasm, which appears translucent, is almost entirely occupied by a relatively thick, dense, orange granulation (0.5–1.5 µm) that, unlike basophils, does not overlap the nucleus. Vacuoles are not typically observed (Figure 1A,B).

Regarding cytochemical aspects, the presence of myeloperoxidase in normal eosinophils is demonstrated through enzymatic cytochemistry and an appropriate substrate. Indirectly, the “content” of myeloperoxidase can be deduced through staining with Sudan Black B (Figure 1C,D). Neutrophils and eosinophils can be distinguished by the thicker granulation of eosinophils. The eosinophil exhibits an absence of esterases and both acid and alkaline phosphatases. The Periodic Acid–Schiff (PAS) reaction is negative, or with weak intergranular positivity, in contrast to the marked positivity of segmented neutrophils. Additionally, metachromasia with Toluidine Blue, which is characteristic in basophil granulocytes, is not observed [3].

**Figure 2:**
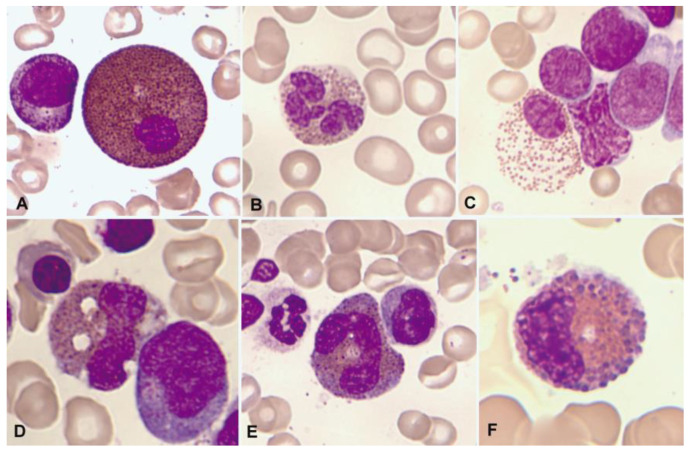
Bone marrow aspirate. May–Grünwald–Giemsa (MGG), ×1000. Dysplastic eosinophils. (**A**) Giant form with a small non-lobulated nucleus. (**B**) Nuclear hypersegmentation. (**C**) Mature eosinophil with a round non-lobulated nucleus and hypogranularity. (**D**) Vacuolar images. (**E**) Persistence of cytoplasmic basophilia in a large band eosinophil. (**F**) Metamyelocyte with thick pre-eosinophilic granulation arranged peripherally.

Morphological alterations of the eosinophil, regardless of whether eosinophilia is reactive or malignant, can include nuclear morphological abnormalities (absence of lobulation, hypersegmentation, or ring nuclei), defects in the cytoplasm (total or partial degranulation, vacuoles, persistent basophilia, visualization of pre-eosinophilic granulation, or increased thickness of granules), size alterations (anisocytosis, gigantism, or small forms), and maturation disharmonies, which encompass previous defects such as the persistence of cytoplasmic basophilia in elements with advanced maturity [10] (Figure 2).

### 1.4. Eosinophil Flow Cytometry

**Figure 3:**
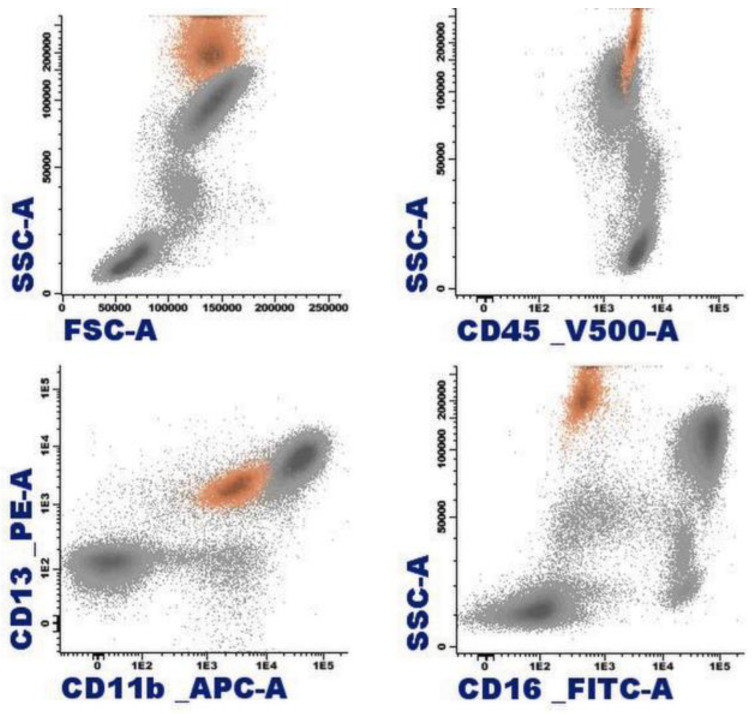
Representation of normal eosinophils (in orange) by flow cytometry in peripheral blood.

By immunophenotype, the normal eosinophil is characterized by a pattern of elevated size (FSC) and complexity (SSC), reflecting its cytomorphological features. They originate in the bone marrow from pluripotent hematopoietic stem cells CD34+ CD117+. They express the panleukocytic marker CD45, and with differentiation, they lose the expression of CD34, CD117, and HLADR, acquiring CD11b, CD15, CD65, and cytoplasmic eosinophilic peroxidase (CyEPO). Eosinophils produce this peroxidase, which shares some homology with the myeloperoxidase that is found in neutrophil and monocyte leukocytes [11]. Other markers that are present in these cells include CD13, CD33, CD193 (CCR3), and the alpha subunit of the high-affinity IgE receptor (FcεR1α) [4,12]. In contrast to neutrophils, they exhibit slightly weaker expression of CD15 in the absence of CD16 (Figure 3) [12].

## 2. Eosinophilia

Eosinophils in peripheral blood constitute 0.3–5% of leukocytes, equivalent to 0.05–0.5 × 10^9^/L. Eosinophilia is considered when a patient’s count is >0.5 × 10^9^/L (between 0.5 and 1.49 × 10^9^/L is also known as mild eosinophilia), and hypereosinophilia is considered when it is ≥1.5 × 10^9^/L. The latter has also been subdivided into moderate hypereosinophilia (1.5–5.0 × 10^9^/L) and severe hypereosinophilia (>5.0 × 10^9^/L) [13,14,15]. When hypereosinophilia is observed in association with clinical organ damage, the concept of “hypereosinophilic syndrome” (HES) is established. However, in some myeloid neoplasms such as chronic myeloid leukemia (CML) and certain variants of acute myeloid leukemia (AML), where leukocytosis reaches very high levels, absolute hypereosinophilia is observed, even though the percentage of eosinophils is low (<3%). This does not imply the presence of symptoms resulting from eosinophil-mediated organ damage. In this context, the International Cooperative Working Group on Eosinophil Disorders (ICOG-EO), during its 2021 Working Conference on Eosinophil Disorders and Syndromes, concluded that in these two hematologic malignancies, hypereosinophilia is only considered if there is an absolute blood eosinophilia (≥1.5 × 10^9^/L) and a relative proportion of ≥10% eosinophils in the peripheral blood leukocyte differentials [15].

In a normocellular bone marrow aspirate, eosinophilia is considered when the rate is above 6%. However, in a bone marrow biopsy, eosinophilia has been proposed when the concentration is ≥20% [13,14,15].

On the other hand, based on the underlying etiology and the nature of eosinophils, four categories of eosinophilias can be established: familial (hereditary), secondary (reactive), primary (clonal, neoplastic), and those of unknown significance (Table 1). Clonal eosinophilias are those associated with myeloid or stem cell neoplasms, in which the eosinophil belongs to the malignant clone [16].

**Figure 4:**
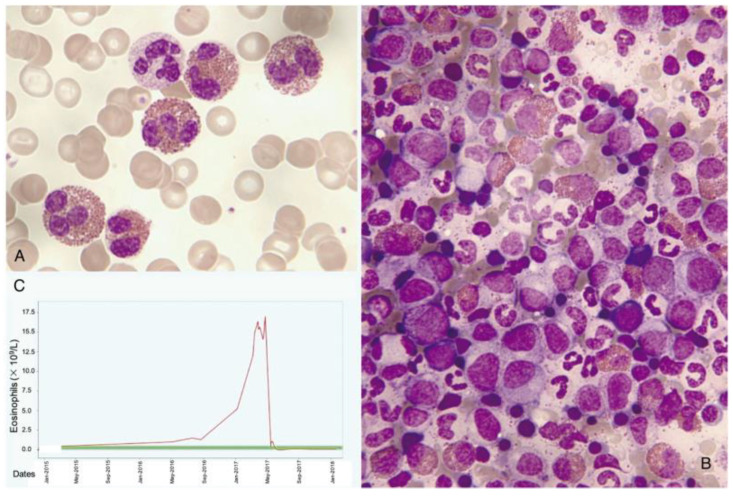
Leukemoid reaction with accompanying hypereosinophilia concurrently with the diagnosis and treatment of cervical cancer. (**A**) Peripheral blood smear showing abundant eosinophils (May–Grünwald–Giemsa [MGG], ×1000). (**B**) Hypercellular bone marrow aspirate with scattered eosinophils (MGG, ×1000). (**C**) Chart displaying elevated eosinophil counts in the blood, which sharply decrease following tumor remission.

In practice, most eosinophilias are secondary (reactive), with the eosinophil being non-clonal. Among their causes are parasitosis, infections, allergic diseases, drugs, chronic inflammatory processes, etc. Solid tumors that can present with a leukemoid reaction featuring blood and/or bone marrow hypereosinophilia, mimicking a myeloproliferative neoplasm (MPN) (Figure 4), should also be included. In a similar manner, some hematological disorders (lymphoid) are associated with eosinophilia that is secondary to the release of eosinopoietic cytokines [16]. Also, some of the new antitumor immunotherapies can cause eosinophilia with dysplasia [17,18].

In the absence of familial and reactive causes, and with clonality ruled out, when eosinophilia is persistent and ≥1.5 × 10^9^/L, the diagnosis is hypereosinophilia of unknown significance. When symptoms due to organ involvement are also observed, it would be classified as idiopathic HES. Organ damage can also occur in the context of the other three groups of eosinophilias, where HES would be, respectively, classified as familial, secondary (reactive), and primary (neoplastic). Regardless of the category, the symptoms overlap in all cases of HES. In this way, clinically relevant eosinophil-mediated organ damage can be considered fibrosis (e.g., in the lungs, heart, digestive tract), thrombosis, skin lesions, central or peripheral neuropathy, vasculitis, and involvement of other organs (liver, pancreas, kidney) [15]. In this context, the use of drugs such as corticosteroids, cytotoxic agents (hydroxyurea, methotrexate, cyclophosphamide), or immunomodulators (interferon-α, cyclosporine) may be necessary. More recently, new eosinophil-targeted therapies have become available, such as monoclonal antibodies targeting IL-5 (mepolizumab and reslizumab) or its receptor (benralizumab). Other biologics in development include dupilumab (targeting the IL-4 receptor) and lirentelimab (targeting Siglec-8), as well as the novel eosinophil-targeting agent, dexpramipexole, with an unknown mechanism of action [19,20].

In clinical practice, the differentiation between reactive eosinophilia (associated or not with hematologic disorders) and neoplastic eosinophilia should be supported by family history, clinical data, laboratory analyses, and imaging tests (Table 1). Additionally, a thorough hematologic work-up, including peripheral blood and bone marrow examination with cytology, flow cytometry, histopathology, cytogenetics/fluorescence in situ hybridization (FISH), and polymerase chain reaction (PCR) should be carried out in order to rule out clonality. Other molecular techniques that can detect clonality and/or complement the aforementioned techniques are SNP-array, next-generation sequencing, nanopore sequencing, and optical genome mapping [21,22,23].

Among the clonality markers described in myeloid hematologic neoplasms or stem cell disorders that are associated with eosinophilia are tyrosine kinase gene rearrangements and *JAK1*, *JAK2*, and *STAT5B* mutations [24,25]. The minimum variant allele frequency (VAF) to consider a variant to be potentially pathogenic in a clonal (non-reactive) eosinophilia is 3% (>2%) [15,26].

Some activating *STAT5B* mutations, particularly the N642H mutation, have recently been identified as recurrent in myeloid neoplasms with eosinophilia. In a study of 1715 patients with eosinophilia, 27 cases were detected, representing 1.6%. Among the myeloid neoplasms showing this mutation, the presentation as chonic eosinophilic leukemia (CEL) is notably significant, observed in 12 out of a series of 82 patients (15%). In CEL, *STAT5B* N642H is dominant or codominant with a high VAF, probably being a driver mutation. In CEL and other myeloid neoplasms, it has been demonstrated by fluorescence-activated cell sorting (FACS) that this mutation is present in eosinophils. On the other hand, *STAT5B* mutations have also been described in T-cell lymphomas with eosinophilia, and it was demonstrated by FACS that the mutation is in the T-cell population and not in eosinophils. *STAT5B* N642H has also been noted in eosinophils from pediatric hypereosinophilias, with or without a presence in the T-cell population. The prognostic value of this mutation and its potential role as a targeted therapy are yet to be determined [25,27,28,29,30,31]. In practice, clonality is not directly determined based on purified eosinophils. Instead, the evidence is indirect and is based on the underlying pathology and identified markers of clonality.

## 3. Hematological Neoplasms Associated with Eosinophilia

Hematological neoplasms that are associated with eosinophilia are divided into two groups: hematological neoplasms that are associated with reactive eosinophilia and hematological malignancies that are associated with neoplastic or primary eosinophilia (Table 2).

### 3.1. Hematological Neoplasms Associated with Reactive Eosinophilia

#### 3.1.1. Classical Hodgkin Lymphoma

**Figure 5:**
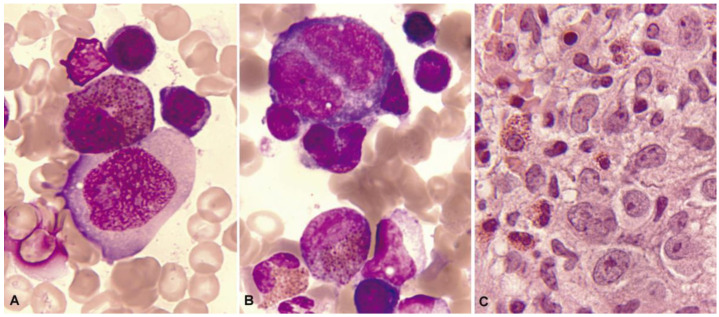
Bone marrow aspirate. May–Grünwald–Giemsa, ×1000. Patient with Hodgkin’s lymphoma presenting infiltrative nodules in the bone marrow with an eosinophilic component. (**A**) Hodgkin cell with a large irregular nucleolus. (**B**) Reed–Sternberg-like cell with mirror nucleus. (**C**) Bone marrow biopsy. Hematoxylin and eosin, ×630. Mononuclear cells with prominent nucleoli and infiltration of eosinophils.

The observation of pronounced and persistent reactive eosinophilia is an unusual finding in patients with B-cell lymphomas, except for Hodgkin lymphoma. Approximately 15% of these patients exhibit it, typically in a mild form. Histologically, it is better distinguished in the inflammatory infiltrate surrounding Reed–Sternberg cells (Figure 5). Inside these cells, IL-5 mRNA has been demonstrated by in situ hybridization. It is unknown whether these cells or the abundant Th2 lymphocytes that are present are the main source of eosinopoietic cytokines [32,33].

#### 3.1.2. Mature T-Cell Neoplasms

Mature T-cell neoplasms that are associated with eosinophilia mostly derive from memory CD4+ T cells, which can produce eosinophilopoietic cytokines. Sometimes it is doubtful whether eosinophilia is due to the tumor cells themselves or to locally attracted reactive Th2 lymphocytes. In any case, proliferating eosinophils are not related to the malignant clone, and can be integrated into the inflammatory context that surrounds the tumor cells. In the setting of eosinophilia, the study of the immunophenotype of T lymphocytes is relevant, with the antibody against TRBC1 JOVI-1 having recently been incorporated and validated as a useful marker in the identification by flow cytometry of clonal T populations with a TCR alphaBeta [34].

**Figure 6:**
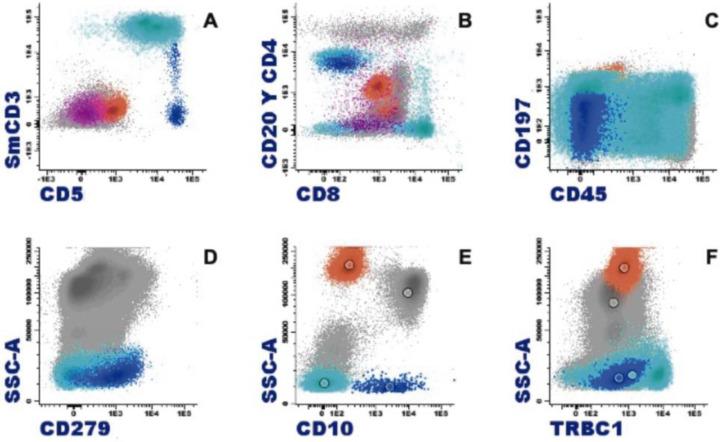
Characteristic immunophenotype of angioimmunoblastic T lymphoma (AITL) with eosinophilia is illustrated in a representative case: light blue, T cells; blue, abnormal T cells; orange, eosinophils; purple, plasma cells. (**A**) Abnormal T cells express CD5 brightly and lack CD3 or show dim expression. (**B**) CD4+ CD3−/+ weak subset. (**C**) Differentiation markers of T cells show effector memory phenotype (CD45RA− CD197−/+ weak). (**D**) Positive expression of CD279. (**E**) CD10 specific pattern of AITL. (**F**) Abnormal T cells show a clonal pattern according to the expression of TRBC1 (TRBC1-negative).

The presence of eosinophilia is estimated in 17–25% of mycosis fungoides cases, and it is described with much higher frequency in Sezary syndrome, where it is observed in 10 out of 13 patients (77%) [32,35]. In both mycosis fungoides and Sezary syndrome, clonal T lymphocytes commonly exhibit a CD4+ CD3+ phenotype in the absence of CD7 and CD26 expression. In the case of Sezary syndrome, a phenotype of central memory CD4 T lymphocytes (CD45RA− CD45RO+ CCR7+ CD27+, and CD62L+/−) is characteristic, unlike mycosis fungoides where its immunophenotype profile is that of an effector memory cell that is resident in the skin (CD45RA− CD45RO+ CCR7− CD27−, and CD62L−). Furthermore, it is accompanied by the expression of intense CD28 and CD279 [36]. In nodal T-follicular helper cell lymphoma, angioimmunoblastic-type eosinophilia is also common, occurring in 32–50% of cases. This lymphoma originates from a follicular CD4 T cell with a phenotype that is characterized by a weak or negative expression of CD3, with expression of CD10 and PD-1 (CD279) maintaining the expression of other pan-T markers such as CD5 (Figure 6) [37]. Adult T-cell leukemia/lymphoma follows in frequency, showing eosinophilia in 20% of cases. In this case, it is generally a CD4+ CD8- T lymphocyte phenotype, although to a lesser extent, they can be double-positive (CD4+ CD8+) or CD4− CD8+. Furthermore, the expression of CD25 and CCR4 is typical, the latter being essential, as it is currently a therapeutic target [38]. More rarely, eosinophilia is found to be associated with other T lymphomas, although histologically, it is frequently found with a peritumoral arrangement [32].

#### 3.1.3. Lymphocytic Variant of Hypereosinophilic Syndrome 

This is a rare variant of hypereosinophilic syndrome (HES), with eosinophilia due to overproduction of eosinophilopoeitic cytokines secreted by clonal T lymphocytes with an abnormal immunophenotype. Persistent blood hypereosinophilia is related to tissue infiltration and organ damage. In a large review of 148 cases, the median age at diagnosis was 46 years, and there was no difference by gender. The most common clinical manifestations were cutaneous (81%), while cardiovascular manifestations occurred in 11.5% of cases. The most frequent immunophenotype of T cells was CD3^−^ CD4^+^. Although their behavior is indolent, they have an increased risk of transformation to T lymphoma (risk of transformation at 10 years: 19.9%) [39].

#### 3.1.4. B-Lymphoblastic Leukemia/Lymphoma

**Figure 7:**
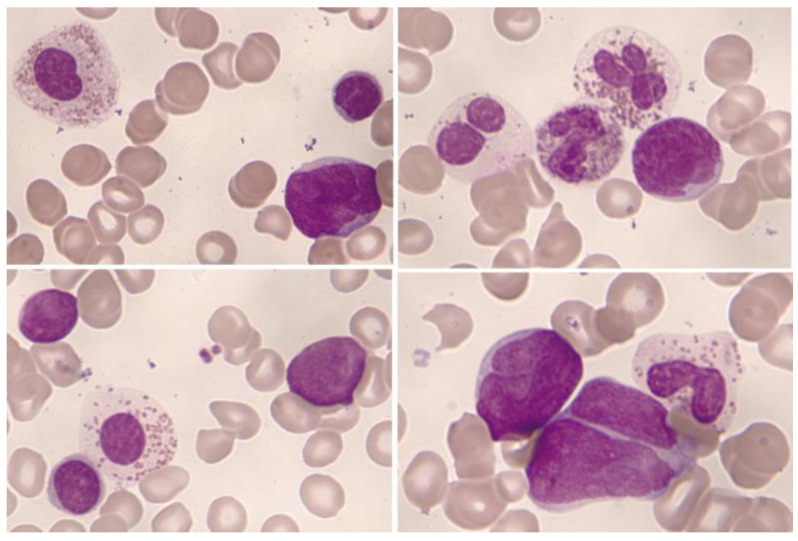
Bone marrow aspirate. May–Grünwald–Giemsa, ×1000. B-acute lymphoblastic leukemia. Blasts accompanied by dysplastic eosinophils characterized by their large size, hypogranularity, and absence of nuclear lobulation.

**Figure 8:**
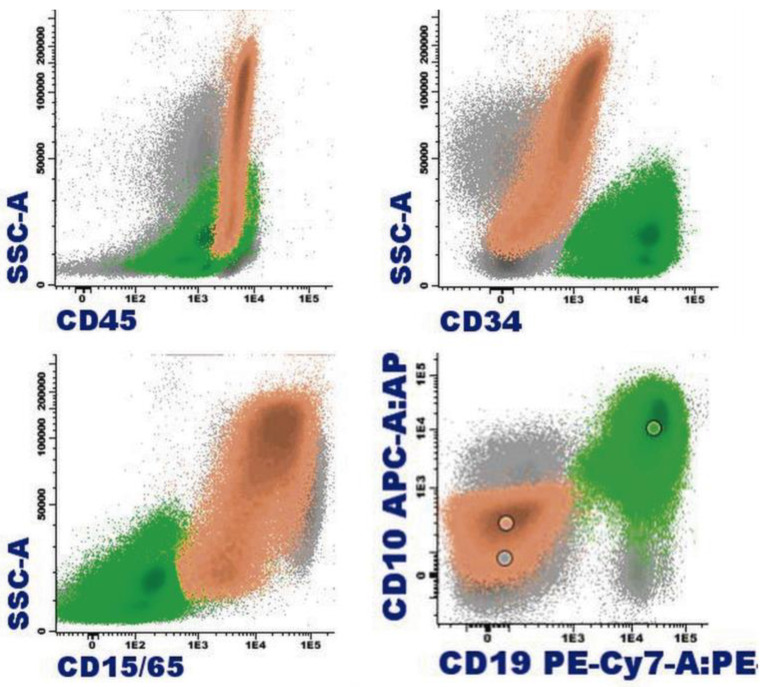
B-acute lymphoblastic leukemia with eosinophilia by flow cytometry (bone marrow): In orange, eosinophils are represented with varying degrees of granulation, mostly hypogranular, making identification challenging (heterogeneous SSC, mostly low). Eosinophils are characterized by intense expression of CD45 and are weakly positive for CD15; the immature B-cell population, highlighted in green, corresponds to a common B-cell acute lymphoblastic leukemia, characterized by weak expression of CD19, CD34, and CD45; positive expression for CD10; and negative expression for CD15/CD65.

Significant eosinophilia during or prior to diagnosis is observed in less than 1% of B acute lymphoblastic leukemia/lymphoma (B-ALL). The expansion of eosinophils is considered to be reactive to the production of cytokines by lymphoblasts. Eosinophils can show dysplastic features in their morphology and by immunophenotype (Figure 7 and Figure 8).

Eosinophilia is often associated with B-ALL with *IGH*::*IL3* fusion [according to the World Health Organization (WHO) nomenclature 2022] or B-ALL with t(5;14)(q31.1;q32.3)/*IL3*::*IGH* [in accordance with the International Consensus Classification (ICC) 2022] [40]. It is a very rare subtype of B-ALL. In a review of 24 patients, this leukemia mainly affects adolescent or young adult males who suffer frequent manifestations derived from eosinophilia (neurological, thromboembolic, pulmonary, and cutaneous) [41]. The relevance of eosinophilia in the blood and bone marrow can relativize the percentage of blasts, which can be less than 20%. In this way, the diagnosis of leukemia when associated with hypereosinophilia may be difficult due to the low fraction of leukemic cells in the bone marrow, within the context of the hypereosinophilic component. In addition, eosinophilia and *PAX5* rearrangement (*PAX5*::*GSDMA* and *PAX5*::*ZCCHC7*) have also been found [42,43], as well as the observation of a hyperdiploid karyotype with a structural alteration [44].

#### 3.1.5. T-Lymphoblastic Leukemia/Lymphoma 

The appearance of eosinophilia in T acute lymphoblastic leukemia/lymphoma (T-ALL) is anecdotal, having been described in a case of near-early T precursor ALL with a t(5;7)(q31;q21)/*CDK6*::*IL3*, which may have a similar functional mechanism to the *IGH*::*IL3* rearrangement of B-ALL [45].

### 3.2. Hematological Malignancies Associated with Neoplastic or Primary Eosinophilia

#### 3.2.1. Myeloid/Lymphoid Neoplasms with Eosinophilia and a Defining Gene Rearrangement

The 2016 WHO classification recognized four entities with genetic rearrangements of *PDGFRA* (4q12), *PDGFRB* (5q32), *FGFR1* (8p11.2), and, as a provisional entity, *PCM1*::*JAK2* [t(8;9)(p22;p24.1)] [46]. Many of these patients had a variable degree of peripheral eosinophilia. In 2022, in the WHO classification, this category was termed “Myeloid/lymphoid neoplasms with eosinophilia (M/LN-eo) and defining gene rearrangements” and “Myeloid/lymphoid neoplasms with eosinophilia and tyrosine kinase gene fusions” (M/LN-eo with TK-gene fusions) in the ICC 2022. Both classifications include a modification in the *PCM1*::*JAK2* category that is now called *JAK2* rearrangement. Also, the latest classifications include three new entities: M/LN-eo with *FLT3* rearrangement, M/LN-eo with *ETV6*::*ABL1* fusion, and M/LN-eo with other tyrosine kinase gene fusions. This group of neoplasms has a very low incidence, with the cell of origin being a pluripotent myeloid/lymphoid stem cell; hence, the great heterogeneity in the involvement of hematopoiesis that is found.

The clinical manifestations, as well as the findings on physical examination, are protean. The most common presentation is as a chronic process resembling myeloproliferative neoplasm (MPN) or myelodysplastic/myeloproliferative neoplasm (MDS/MPN). In this chronic phase, only a constitutional syndrome may be observed, occasionally with hepatosplenomegaly. Less frequently, M/LN-eo presents with increased aggressiveness and clinical deterioration, either as an initial manifestation or as progression from a chronic phase, in the form of AML, T-ALL with lymphadenopathy, and more rarely, as B-ALL or acute leukemia of ambiguous lineage. In some patients, the same neoplasia occurs simultaneously and chronically in the bone marrow and aggressively in the extramedullary involvement [47,48]. The pathophysiology of these diseases includes the expression of a fusion gene that involves *PDGFRA*, *PDGFRB*, *FGFR1*, *JAK2*, *FLT3*, *ABL1*, or other kinases, giving rise to an aberrant constitutively activated tyrosine kinase.

No cases of M/LN-eo have been described as low-grade B-cell lymphoid neoplasms. Singularly, when the clinical presentation is a B-ALL, the differential diagnosis must be made with B-ALL with *BCR*::*ABL1*-like features. In the latter case, rearrangement is restricted to lymphoblasts and typically is not accompanied by eosinophilia [48]. 

The observation of blood, bone marrow, and in certain cases, extramedullary eosinophilia, is often a common sign that guides the diagnosis, although occasionally peripheral or tissue expression may be absent or very mild. In most patients, eosinophilia is typically moderate, although it can reach the level of hypereosinophilia with potential organ infiltration, especially in cases with CEL morphology. When its presentation is as MPN or MDS/MPN, a certain degree of fibrosis in the bone marrow is frequently observed. 

The vast majority of rearrangements can be diagnosed by FISH and suspected in cytogenetic study, except for *FIP1L1*::*PDGFRA*, which is cryptic. At diagnosis, reverse transcription polymerase chain reaction (RT-PCR) can also be used for the *FIP1L1*::*PDGFRA* rearrangement. It would be advisable to know the partner in these fusion genes so that RT-PCR can be used to follow up. Exceptionally, in all groups, there may be cryptic cases that require other molecular techniques such as next-generation sequencing (including RNA sequencing), nanopore sequencing, or SNP-array for diagnosis. Optical genome mapping is proving to be a good alternative in the diagnosis of these rearrangements [21,22,23].

Significantly, the majority of patients are male, and the illness occurs at any stage of life [47,48,49,50,51]. The main characteristics of the different categories of M/LN-eo with eosinophilia are summarized in Table 3.

##### Myeloid/Lymphoid Neoplasm with *PDGFRA* Rearrangement

Platelet-Derived Growth Factor Receptor-α (*PDGFRA*) is a gene that is located on 4q12 and that encodes the synthesis of a tyrosine kinase (TK) receptor protein involved in cell division and survival. M/LN-eo with *PDGFRA* rearrangement is the most common M/LN-eo. The annual incidence has been estimated at around 0.18 cases per million inhabitants [52]. The *PDGFRA* rearrangement that is by far the most commonly observed is the *FIP1L1*::*PDGFRA* fusion, a cryptic translocation due to an interstitial deletion in 4q12. This category also includes variant translocations of *PDGFRA* with other genes such as *KIF5B* [53], *CDK5RAP2* [54], *STRN*, *ETV6* [55], *BCR* [56], *TNKS2* [57], and *FOXP1* [58]. Although somatic mutations may also exist in this group, it does not seem that they have a clear effect on event-free survival [59].

At diagnosis, it usually presents as MPN, almost always as chronic eosinophilic leukemia, with the eosinophils frequently being dysmorphic, although they can be practically normal. Eosinophilia is a generalized trait (close to 100%), although there may be a selection bias in some series that only included patients with eosinophilia. However, isolated cases lacking relevant eosinophilia have been described [60]. Generally, the bone marrow is hypercellular, with an increase in eosinophils that can be normal or dysmorphic; there may also be an increase in blast (<20%), and an increase in dispersed mast cells is common or, more rarely, it forms loose clusters, which may present an atypical spindle-shaped morphology and usually express CD25. Histologically, the finding of moderate reticulin fibrosis is characteristic [52].

In certain cases, this chronic phase may undergo clinically aggressive blastic progression, primarily to AML or T-ALL, exceptionally to B-ALL [61,62]. Occasionally, a chronic bone marrow presentation may be accompanied by extramedullary involvement due to a more aggressive process [63].

Less common are presentations as acute leukemia, generally with peripheral eosinophilia. Its presentation as B-ALL is not described except for a single case within a series of cases of Ph-like B-ALL [64]. Although infrequently, a minimum of cases could present with extramedullary involvement without concomitant bone marrow involvement [47]. Anecdotal presentations include a patient with three synchronous hematologic malignancies driven by the *FIP1L1*::*PDGFRA* rearrangement: cutaneous T lymphoma, T-lymphoblastic lymphoma in lymph node, and MPN in bone marrow [65]. Likewise, some association with papulosis lymphomatoid has been described [66].

Within *PDGFRA* rearrangements, the series by Rohmer et al. stands out, comprising 151 patients in the chronic phase gathered in a retrospective study. The mean age at diagnosis was 49 years (±12), and 95% were male. Only 17% of the patients were initially asymptomatic; the majority of the remaining individuals presented constitutional symptoms, and up to 72% exhibited at least one symptom that was related to hypereosinophilia; approximately half of the cases showed splenomegaly. In lab tests, all patients presented eosinophilia, and in up to 31%, this was the only finding. Anemia (24%), mild thrombopenia (28%), and variable leukocytosis, often with neutrophilia (20%) but also with monocytosis (16%) or basophilia (13%), are also described. The serum levels of vitamin B_12_ and tryptase were regularly elevated, being an increase in IgE much less common [52]. Another series of interest is the German Registry for Disorders of Eosinophils and Mast Cells (GREM), in which 78 cases are described with a median age of 45.5 years (range of 19–70). Sixty-five patients (83%) were diagnosed in the chronic phase, of whom four experienced an aggressive evolution during follow-up, and thirteen cases (17%) were diagnosed in the blastic phase. Fourteen out of the seventeen cases that developed acute leukemia were of the myeloid lineage [49].

In pediatric ages, this rearrangement is extremely rare, without a defined sex predominance, although they are predominantly MPN with eosinophilia type CEL [63].

Virtually all patients with *PDGFRA* rearrangements respond to imatinib, with a sensitivity that is 100 times greater than *BCR*::*ABL1*, which emphasizes the importance of early diagnosis [51,52,67,68].

##### Myeloid/Lymphoid Neoplasm with *PDGFRB* Rearrangement

Platelet-Derived Growth Factor Receptor-β (*PDGFRB*) is a gene that is located on 5q32 and that encodes the synthesis of a tyrosine kinase receptor protein that is involved in the mitogenic activity of mesenchymal cells. *PDGFRB* rearrangement follows in order of frequency to the *PDGFRA* rearrangement. Both types of neoplasms share a marked parallelism in terms of male predominance (although it is less pronounced and more variable according to the series in *PDGFRB*-rearranged cases [48,49,69,70,71]), median age of presentation in adults, clinical and cytological presentation, and in their excellent response to imatinib [51,69]. This group of patients often presents with prominent eosinophilia (hypereosinophilia in 50–58% of cases), although eosinophilia may be absent in up to 21–25% of patients in some series; and monocytosis is also frequent [49,69]. Organ involvement is not significant, except for splenomegaly, which is described in up to 83% of affected patients and which may be accompanied by hepatomegaly. Eosinophil-mediated organ damage is possible, since patients can present with hypereosinophilia and also CEL [69].

**Figure 9:**
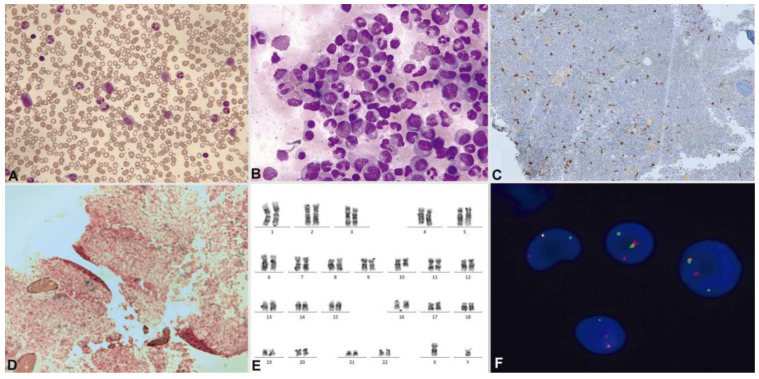
Patient with M/LN-eo with *PDGFRB* rearrangement (courtesy or Dr. Marina Gómez Rosa, Virgen de Valme hospital, Seville). May–Grünwald–Giemsa. (**A**) Peripheral blood: myelemia and left shift. (**B**) Hypercellular bone marrow (aspirate) with eosinophilia. Bone marrow biopsy: (**C**) immunohistochemistry with CD117 reveals an increase in scattered mast cells. (**D**) Bone marrow biopsy showing Grade 1 reticulin fibrosis by the World Health Organization criteria. (**E**) Bone marrow karyotype: 46,XY,t(5;10)(q32;q21) [20]. (**F**) Interphase FISH using a *PDGFRB* break-apart probe: *PDGFRB* rearrangement appears as a split red/green signal.

More than 40 different fusion partners of *PDGFRB* have been described, with *ETV6* being the most frequent [72,73,74,75,76,77]. In this case, a presentation as chronic myelomonocytic leukemia (CMML) with eosinophilia and t(5;12)(q32;p13.2) is typical. Most *PDGFRB* rearrangements are detected by alterations in the karyotype that affect 5q32 and by FISH, although exceptionally, this rearrangement can be cryptic and require RNA sequencing or alternative approaches for diagnosis. Somatic mutations are also described in this group, without a clear effect on event-free survival [59]. The high availability of partners seems to influence the heterogeneity of presentation in the chronic phase. In this way, *PDGFRB*-rearranged cases may present as MPN or MDS/MPN with eosinophilia (more like CMML and less like atypical chronic myeloid leukemia) (Figure 9). M/LN-eo with *PDGFRB* rearrangement can also present as myeloid or lymphoid acute leukemia. In exceptional cases, progression as angioimmunoblastic T cell lymphoma has been described [69].

In a series of 135 adult patients with M/LN-eo, 26 cases corresponded to the subcategory of *PDGFRB* rearrangements. Of these cases, 22 (85%) presented as chronic phase, while 4 cases (15%) were in the blast phase, with 1 patient developing a blast phase during follow-up [49]. At least nine pediatric cases have been reported, with no differences by gender [78,79,80,81,82,83].

##### Myeloid/Lymphoid Neoplasm with *FGFR1* Rearrangement

The *FGFR1* (Fibroblast Growth Factor Receptor 1) is a gene that is located on 8p11 which plays a role in the synthesis of a tyrosine kinase protein involved in mitosis, development, and maturation of cells. The cases with this rearrangement are particularly rare, with around 100 cases having been reported. As with the previous subtypes, the presentation is highly heterogeneous. However, the common link is the demonstration of the rearrangement of *FGFR1* (chromosome 8p11). These rearrangements are usually observed by cytogenetics, although there are cryptic cases that are detected by FISH and/or RNA sequencing [84]. At least 14 partner genes have been reported. The most frequently observed are *ZMYM2* in t(8;13)(p11;q12), followed by *BCR*, *CEP110*, and *FGFR1OP* (*FOP*). While a certain tendency for the “partner” to influence the phenotype of the disease has been observed, this is not always the case. It is the group that has the highest frequency of mutations, especially in *RUNX1*, which could contribute to an inferior outcome [59]. The age range of the patients is wide (1–87 years), with a median age in adults in the largest series between 46 and 51 years [85,86]. Unlike *PDGFRA* and *PDGFRB* rearrangements, men displayed only a slight predominance of *FGFR1* rearrangement—slightly above 50% [85,86,87].

M/LN-eo with *FGFR1* being rearranged presents most frequently as MPN with or without concomitant involvement by lymphoblastic lymphoma or acute leukemia and less like a myeloid, lymphoid, or mixed-lineage disease in the blastic phase, which can also be only extramedullary. Blood eosinophilia is common, ranging from 50% to 80% (assessed in short series of patients) [48,49,86]. A high tendency towards blastic transformation (AML, B or T-ALL, or mixed-phenotype leukemia) was observed [88,89,90]. 

Presentations have been described as B-ALL with *BCR*::*FGFR1* rearrangement and additional cytogenetic alterations. When acute leukemia is treated and remitted, MPN appears with leukocytosis, splenomegaly, and isolated persistence of the *FGFR1* rearrangement [89].

About 27 cases are described with this rearrangement that present as acute leukemia of a mixed phenotype, ambiguous lineage or switching lineage in the evolution (8 cases), without eosinophilia in up to 21% of the cases [84]. 

In children, at least seven cases have been published [82,91,92,93,94,95,96].

A highly significant difference of this group of patients compared to *PDGFRA* and *PDGFRB* rearrangements is their lack of response to first- and second-generation tyrosine kinase inhibitors. The clinical course of the *FGFR1* rearrangement cases is usually aggressive, with rapid progression to blast crisis and a short period of patient survival. Aggressive chemotherapy and allogeneic stem cell transplantation are considered the best curative options. In isolated cases, clinical trials with other therapeutic targets, such as pemigatinib, ponatinib, sorafenib, or olverembatinib, have demonstrated some clinical efficacy [92,97,98,99,100,101].

##### Myeloid/Lymphoid Neoplasm with *PCM1*::*JAK2* Rearrangement

The *JAK2* gene is located on 9p24 and gives rise to the synthesis of a tyrosine kinase protein that promotes cell proliferation. In numerous Ph-negative myeloproliferative neoplasms, a specific somatic mutation, V617F, is demonstrated in the *JAK2* gene. However, chromosomal translocations involving the *JAK2* gene are infrequent and occur in various hematologic neoplasms. The *PCM1*::*JAK2* fusion gene arising from t(8;9)(p22;p24) is the most common. This rearrangement involves numerous hematopoietic lineages, giving rise to chronic or blastic hematological malignancies with varying degrees of eosinophilia. *JAK2* can also rearrange with *ETV6* or *BCR*, exhibiting a similar clinical behavior. Thus, in the fifth edition of the WHO Classification and the ICC, this subtype has been redefined as M/LN-eo with *JAK2* rearrangement [26,102]. Somatic mutations have been reported in this group, but they are much less frequent than in cases with *FGFR1* rearrangement and more similar to cases with rearranged *PFGFRA* and *PDGFRB* [48,59]. To date, approximately 100 cases of M/LN-eo with *PCM1*::*JAK2* rearrangement or variants have been described. In a review of 66 cases with *PCM1*::*JAK2* rearrangement [103], the mean age of presentation was 47 years (range, 6–86 years), with 77% being men. The most common presentation was chronic, as MPN or MDS/MPN, with eosinophilia in 75% of patients, and often with erythroid proliferation dysplastic. Other less frequent presentations were as acute leukemia or blast crisis from a previous MPN, or a minority as cutaneous T-cell lymphoma. When this subtype presents as B-ALL, a differential diagnosis must be made with Ph-like B-ALL, although rearrangements with certain partners (*SSBP2*, *PAX5*, *RFX3*, *USP25,* and *ZNF274*) are normally considered Ph-like B-ALL. Survival is highly variable, depending on presentation, with worse outcomes in the blastic forms. The limited number of patients, their clinical heterogeneity, and the short follow-up make the choice of therapeutic approaches challenging. Targeted therapy with *JAK2* inhibitors, such as ruxolitinib, may be beneficial by inducing hematologic or molecular improvement of benefit [77,104,105], but allogeneic stem cell transplantation is considered the only curative option [50].

##### Myeloid/Lymphoid Neoplasms with Eosinophilia and Defining Gene Rearrangement: New Entities

In 2022, both the fifth edition of the WHO Classification and the ICC recognized the following three new groups within the M/LN-eo and tyrosine kinase (TK) fusion genes category:

1. Myeloid/lymphoid neoplasms with *FLT3*/t(v;13q12.2) rearrangements

*FLT3* is a class III tyrosine kinase receptor that is involved in the regulation, differentiation, proliferation, and survival of hematopoietic progenitor cells. The unusual finding of hematological neoplasms accompanied by eosinophilia with rearrangements of *FLT3* in multiple hematopoietic lineages has allowed for the differentiation of a new category of M/LN-eo. Just over 30 cases have been described.

In a multicenter study including 12 patients and case report descriptions, the most common partner was *ETV6* (12p13), followed by isolated cases of other partners such as *ZMYM2*, *TRIP11*, *SPTBN1*, *GOLGB1*, *CCDC88C*, *MYO18A*, and *BCR* [106,107]. There may be additional somatic mutations, but not in *FLT3* [106]. Although the presentations were diverse, often manifesting as chronic processes, and the age range varied widely, they commonly shared a predominance in males and frequent eosinophilia in blood, bone marrow, or tissues [106,108]. Extramedullary involvement is common in cases in the blastic phase, with the coexistence of bone marrow involvement due to chronic processes and extramedullary involvement due to blastic processes having been described [48].

Pediatric cases are anecdotal, but at least three cases have been described [106,109,110].

*FLT3*/t(v;13q1212) rearrangements are largely detectable by cytogenetics (chromosome 13q12) but can be cryptic, which would require FISH and other molecular techniques such as RNA sequencing. The importance of their recognition lies in the fact that they respond to *FLT3* inhibitors. In any case, allogeneic stem cell transplantation could be a long-term survival option for these patients [106,109,111,112,113].

2. Myeloid/lymphoid neoplasms with *ETV6*::*ABL1*/t(9;12)(q34.1;p13.2) fusion

The *ABL1* gene, located on 9q34.12, encodes a tyrosine kinase protein that plays a role in the regulation of apoptosis and cell proliferation. This category currently only includes one *ABL1* partner, which is *ETV6*. It has been reported in MPN, acute lymphoblastic leukemia (B and T), and AML. Although at least 13 other partners of *ABL1* have been described, they correspond to Ph-like B-ALL or de novo T-ALL [114,115]. 

Most of these rearrangements are produced by an insertion of either *ABL1* in *ETV6* or *ETV6* in *ABL1*. A translocation t(9;12) is less frequent, since both genes would be transcribed in the opposite direction in this case, and an inversion of one of the two genes would also be required. Thus, its detection by cytogenetics is usually cryptic, and it is diagnosed by FISH with break-apart probes for *ABL1* (more useful when the insertion is of *ABL1* in *ETV6*) and for *ETV6* (more useful when the insertion is of *ETV6* in *ABL1*). Other molecular techniques such as RNA sequencing can also confirm this rearrangement and provide a diagnosis if it has been previously overlooked. There are no conclusive data on somatic mutations in this group.

Because the *ETV6*::*ABL1* rearrangement shares the constitutive activation of the same tyrosine kinase with the *BCR*::*ABL1* rearrangement, it has the peculiarity of being the one that most closely resembles chronic myeloid leukemia (CML). The myeloid neoplasm with E*TV6*::*ABL1* rearrangement typically presents in the chronic phase with a CML-like morphology. The presence of eosinophilia is almost constant, and basophilia is common. As in CML, there may be progression to blast crisis (myeloid or lymphoid), or rarely, patients present as AML. When the *ETV6*::*ABL1* rearrangement presents as B-ALL, it most often corresponds to a Ph-like B-ALL.

In the series by Zaliova and collaborators (own and a review of the literature) that includes 44 cases with this rearrangement, 22 cases correspond to myeloid malignancies (18 MPN, 4 AML); and up to 22 cases were ALL (21 being Ph-like ALL by a standardized assay). The median age in the 18 cases described in the chronic phase is 51 (range, 24–72 years), with a predominance in men [116].

In the chronic phase, these patients respond to tyrosine kinase inhibitors, such as imatinib, or second- and third-generation ones. Just like in CML, the response is poor in the blast phase [51,116,117].

3. Myeloid/lymphoid neoplasms with other tyrosine kinase gene fusions

These gene fusions affect other tyrosine kinase genes that are not included in the previous categories, have hematopoietic lineage plasticity and eosinophilia, and present similarly. Some of these rearrangements are *ETV6*::*FGFR2* [118], *ETV6*::*LYN* [119], *ETV6*::*NTRK3* [120], *RANBP2*::*ALK* [121], *BCR*::*RET*, and *FGFR1OP*::*RET* [122]. In these rearrangements, experimental treatment with other tyrosine kinase inhibitors could be eligible [118,119,122,123,124].

#### 3.2.2. Core Binding Factor Acute Myeloid Leukemias

##### AML with inv(16)(p13.1q22) o t(16;16)(p13.1;q22); *CBFB*::*MYH11*

**Figure 10:**
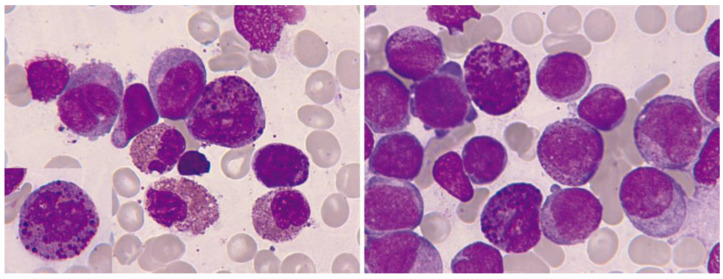
Bone marrow aspirate. May–Grünwald–Giemsa, ×1000. Acute myeloid leukemia with inv(16)(p13.1q22). In both images, dysmorphic eosinophils with thick reddish-violet granules are evident. In the left image, the intensity of eosinophilia is observed.

The diversity of these leukemias presents a myelomonocytic cytology, which is sometimes purely monocytic. The percentage of eosinophils in the blood is usually normal—although eosinophilia may exist, as these leukemias are usually hyperleukocytic—and eosinophils do not present relevant dysplasia. In the bone marrow examination, eosinophilia is usually clear, with immature elements. Thick and dark violet-purple granules are characteristic (Figure 10) [125,126].

**Figure 11:**
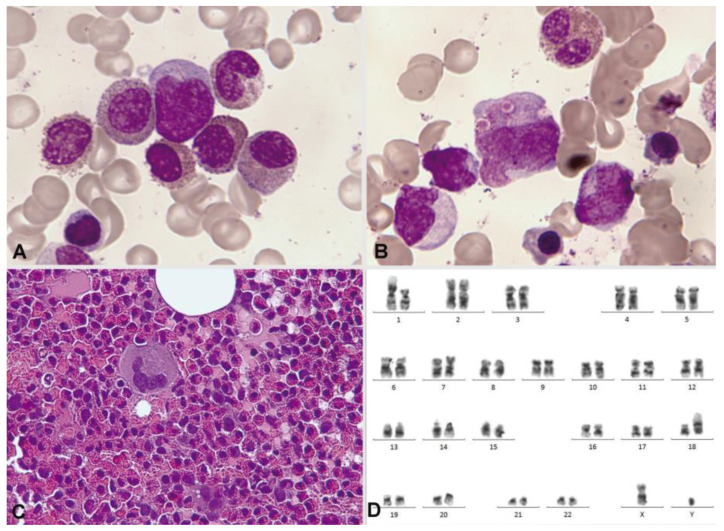
Bone marrow aspirate. May–Grünwald–Giemsa, ×1000. Oligoblastic acute myeloid leukemia with inv(16)(p13.1q22). (**A**) Cytological image with a blast containing a long Auer rod, surrounded by eosinophils. (**B**) Pseudo-Chédiak–Higashi inclusions. (**C**) The histological image clearly shows massive bone marrow eosinophilia. (**D**) Bone marrow karyotype: 46,XY,inv(16)(p13.1q22) [3]/46,idem,t(1;18)(p22;p11.2) [17].

At times, the presence of eosinophils, less than 6%, is observed in a blastic hypercellularity environment. In our series of 13 cases, we observed blood eosinophilia in 6/13 (46%) and marrow eosinophilia in 10/13 (77%). In exceptional cases, bone marrow eosinophilia becomes massive, without expression in the blood (Figure 11).

**Figure 12:**
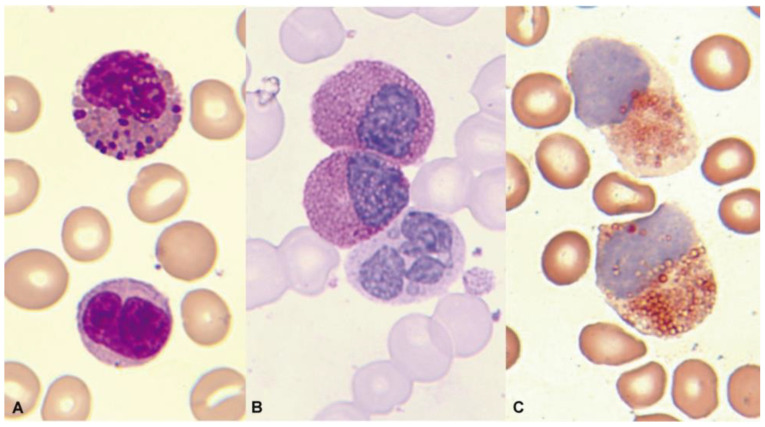
Bone marrow aspirate. May–Grünwald–Giemsa, ×1000. Acute myeloid leukemia with inv(16)(p13.1q22). (**A**) Eosinophil with thick dark pre-eosinophilic granules. (**B**) Positive periodic acid–Schiff (PAS) reaction in immature eosinophils, in contrast to the negativity in an immature segmented neutrophil. (**C**) Two immature eosinophils showing marked chloroacetate esterase activity.

Cytochemically, positivity is typical both with the PAS reaction and in the enzymatic demonstration of chloracetate esterase (Figure 12). In the immunophenotypic pattern, the presence of blasts with myeloid differentiation is common, as is another subtype of blasts with commitment to monocytes according to their markers [127].

##### AML with t(8;21)(q22;q22.1); *RUNX1*::*RUNX1T1*

**Figure 13:**
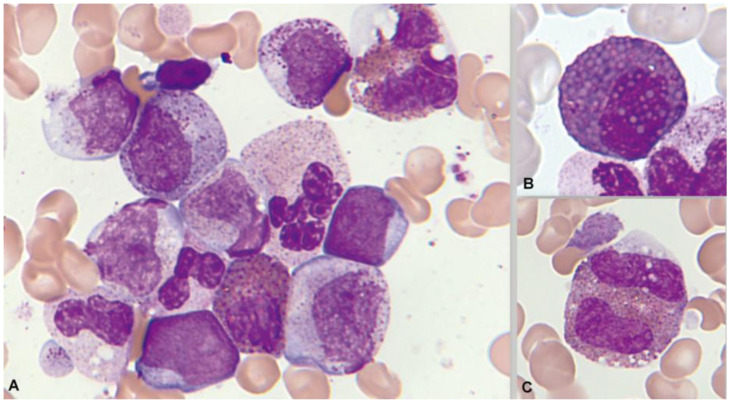
Bone marrow aspirate. May–Grünwald–Giemsa, ×1000. Acute myeloid myeloid with t(8;21)(q22;q22.1). (**A**) Granulocytic dysplasia with peripheral basophilia reinforcement. (**B**) Dysplastic eosinophils with increased granulation thickness. (**C**) Eosinophils with mirror nuclei.

These leukemias are known for their peculiar morphology, with large Auer rods in the blasts and marked neutrophil dysgranulopoiesis. Eosinophilia, both in the blood and in the marrow, is much less evident than in the previous group. In a series of 165 patients collected by the Spanish Hematological Cytology Group [128], blood eosinophilia was observed in 7/148 cases (4.7%) and marrow eosinophilia in 22/137 (16%) (unpublished data). The eosinophils are less dysplastic and without thick and dark red-violet granulation (Figure 13). The chlorocetate esterase is negative, and the PAS reaction may be negative or positive [125,129,130]. In the immunophenotype, most common is the presence of immature blasts with expression of CD34 and CD117, with expression of myeloid markers, such as CD13 and CD33 and lymphoid markers, especially CD19 and CD56 [127].

#### 3.2.3. Mastocytosis

In mastocytosis, a clonal proliferation of eosinophils can be observed that would have its origin in the same neoplastic precursor (multilineage involvement due to the *KIT* mutation).

The 2022 WHO classification includes peripheral and/or central eosinophilia among the signs of myeloproliferation and/or myelodysplasia that are part of the B findings of systemic mastocytosis (SM). However, this is only the case when no reactive cause is identified and the criteria for associated hematological neoplasia are not met [26,131].

In a series of 2350 patients with mastocytosis, eosinophilia was reported in 6.8% and hypereosinophilia in 3.1%, mainly associated with advanced forms. Eosinophilia is frequently seen in bone marrow aspirates and biopsies, even in cases without significant peripheral eosinophilia [132,133]. In exceptional cases, eosinophils can become phagocytosed by the mast cells of an SM, perhaps as a sign of malignancy [134].

In advanced SM with eosinophilia, a differential diagnosis must be made with M/LN-eo and defining gene rearrangements, because the coexistence of both is exceptional [135]. It should be noted that in the M/LN-eo, an increase in mast cells with an aberrant expression of CD25 with or without the expression of CD2 may occur. However, dense multifocal infiltrates do not form, and they do not present *KIT* mutations [26]. Another frequently described marker (80%) in SM mast cells is CD30 [136,137], for which no conclusive descriptions currently exist in the M/LN-eo. To diagnose the presence of both entities, it is necessary to demonstrate the rearrangement of an M/LN-eo and meet the SM criteria [138].

#### 3.2.4. Myeloproliferative Neoplasms

MPN can present with eosinophilia that requires the ruling out of associated MS or M/LN-eo. Within this group, eosinophilia is more common in CML, and especially in CEL.

##### Chronic Myeloid Leukemia

In the chronic phase, eosinophilia may be present in peripheral blood and bone marrow. Unusual presentation forms have been identified, and they are recognized as “eosinophilic variants of CML” with intense eosinophilia, similar to chronic eosinophilic leukemia. Approximately six cases have been described, with a lower median age than that of CML, most without splenomegaly, and with frequent cutaneous manifestations and vascular symptoms [139].

##### Chronic Eosinophilic Leukemia

CEL (2022 WHO), or CEL, not otherwise specified (ICC 2022) is an extremely rare disorder. CEL is accompanied by peripheral hypereosinophilia and significant infiltration of the marrow and different organs. Cases with other MPN or MPN/MDS, M/LN-eo, MDS, mastocytosis, and AML with CBF translocations are excluded. The diagnostic criteria for CEL have been updated in the 2022 WHO classification. In addition to eosinophilia (on at least two occasions over an interval of at least four weeks), the criteria include evidence of clonality and abnormal bone marrow morphology. The WHO has eliminated the increase in blasts (≥5% in the bones marrow and/or ≥2% in the peripheral blood) as an alternative CEL criterion to clonality. This finding is maintained along with the rest of the criteria in the ICC 2022 [26,102].

Organ involvement due to eosinophil infiltration in the absence of abnormal bone marrow morphology, blastosis, and/or genetic clonality points to idiopathic HES. In the same circumstances, if organ damage is absent, idiopathic hypereosinophilia would be considered.

One of the best studied series is from the Mayo Clinic, with 17 patients who were diagnosed according to the 2016 WHO criteria (median 63 years, male 88%). Most patients presented with systemic symptoms due to digestive, cardiac, and pulmonary involvement, as well as splenomegaly and hepatomegaly. The blood count showed leukocytosis with eosinophilia (median 6.4 × 10^9^/L) and moderate anemia. Eosinophils presented dysplasia in half of the cases. In bone marrow, an increase in the M:E ratio 5:1 (47%), eosinophilia (median 43%), dysmegakaryopoiesis (41.2%), fibrosis (17%), and one case with blasts (>5%) was described. Cytogenetic alterations were described in 15/17 patients and mutations in *ASXL1* in 2 patients. The prognosis was poor, with a median survival of 16 months and progression to acute leukemia in only three cases [140].

Activating mutations in *JAK1* and *STAT5B* have also been described as markers of clonality and potentials for targeted therapies in CEL. *STAT5B* N462H is a recurrent mutation in LEC. So, *STAT5B*-mutated eosinophilia/HES should be reclassified as myeloid neoplasm or CEL [24,25,27].

#### 3.2.5. Myelodysplastic/Myeloproliferative Neoplasms

MDS/MPN can present with eosinophilia. In these cases, an associated systemic mastocytosis must be ruled out and/or, less frequently, an M/LN-eo.

#### 3.2.6. Myelodysplastic Neoplasms

The finding of eosinophilia in MDS is unusual and requires reactive causes to be ruled out. A retrospective series of 288 patients described marrow eosinophilia in 36 (12.5%) patients, of which only 18% had peripheral eosinophilia. The usual assumption is that eosinophilia is part of the neoplastic clone. The most common cytogenetic abnormalities are alterations of chromosome 7, complex karyotypes, and isochromosome 17q. A worse survival of these patients is reported, but it could be mediated by the underlying cytogenetics [141].

**Table 3:** Myeloid/lymphoid neoplasms with eosinophilia and defining gene rearrangements (WHO 2022). Myeloid/lymphoid neoplasms with eosinophilia and tyrosine kinase gene fusions (ICC 2022).

M/LN-eo with	Frequency * n/Total Cases (%)	Age Median or Range Median (Age Range)	Male/Female Ratio	Most Common Presentation	Treatments	Patient Series
*PDGFRA* rearrangement	78/135 (57.8%)	Range median 45.5–51 years (2–82)	17.8:1 to 43:1	CEL	Imatinib mesylate	Pozdnyakova et al., 2021 [48] Akiely et al., 2022 [63] Metzgeroth et al., 2023 [49]
*PDGFRB* rearrangement	26/135 (19.3%)	Range median 38–53 years (0.25–86)	2.5:1 to 10:1	MDS/MPN or MPN with eosinophilia	Imatinib mesylate	Metzgeroth et al., 2023 [49] Pozdnyakova et al., 2021 [48] Di Giacomo et al., 2021 [71] Jawhar et al., 2017 [69] Cheah et al., 2014 [70]
*FGFR1* rearrangement	9/135 (6.7%)	Range median 46–62 years (0.41–84)	1.1:1 to 1.6:1	MPN with/without lymphoblastic lymphoma and eosinophilia	Allogeneic stem cell transplantation Intensive chemotherapy Ponatinib Midastaurina Futibatinib Pemigatinib (NCT03011372)	Patnaik et al., 2010 [142] Strati et al., 2017 [85] Hernández-Boluda et al., 2022 [86] Parasuraman et al., 2022 [87] McKeague et al., 2023 [84]
*JAK2* rearrangement	11/135 (8.1%)	Median 47 (6–86)	3.4:1	MPN or MDS/MPN with eosinophilia	Allogeneic stem cell transplantation Inh JAK1/JAK2 ruxolitinib (Phase II clinical trial with Ruxolitinib NCT03801434)	Tang et al., 2019 [143] Pozdnyakova et al., 2021 [48] Kaplan et al., 2022 [103]
*FLT3* rearrangement	Not reported	Median 46.5 (0.6–80)	1.8:1	MPN or MDS/MPN with eosinophilia	Allogeneic stem cell transplantation Sorafenib, sunitinib, or gilteritinib	Tang et al., 2021 [106]
*ETV6*::*ABL1* rearrangement	11/135 (8.1%)	Median 51 (24–72)	2.4:1	CML-like with eosinophilia	Dasatinib or nilotinib Imatinib mesylate	Zaliova et al., 2016 [116]
Other tyrosine kinase gene fusions: *FGFR2* *LYN* *NTRK3* *ALK* *RET*	Not reported	Few cases	Few cases, but predominates in males	Variable	*ETV6*::*FGFR2*: TKI (ponatinib) or FGFR inhibitors *ETV6*::*LYN:* Dasatinib *ETV6*::*NTRK3*: TRK inhibitor *RANBP2*::*ALK:* Crizotinib *BCR*::*RET* and *FGFR1OP*::*RET*: Sorafenib	Isolated cases

M/LN-eo: myeloid/lymphoid neoplasms with eosinophilia; CEL: chronic eosinophilic leukemia; MDS/MPN: myelodysplastic/myeloproliferative neoplasm; MPN: myeloproliferative neoplasm; CML: chronic myeloid leukemia; TKI: tyrosine-kinase inhibitors; TRK: tropomyosin receptor kinase. * Frequency within the M/LN-eo is based on the series by Metgeroth et al., 2023, with 135 cases of M/LN-eo [49].

## 4. Conclusions

Eosinophilia that is associated with a hematological malignancy can be reactive or secondary to the production of cytokines eosinophilopoietic. This type of eosinophilia is mainly observed in lymphoid neoplasms. Eosinophilia can also be neoplastic and primary, derived from the malignant clone, usually in myeloid or stem cell neoplasms.

No data collected in cytological and cytometric studies can predict whether eosinophilia is reactive or clonal. The verification is indirect and supported by the categorization of the accompanying hematological neoplasm.

A thorough hematologic work-up, including peripheral blood and bone marrow examination with cytology, flow cytometry, histopathology, cytogenetics/fluorescence in situ hybridization, polymerase chain reaction, and other molecular techniques, should be carried out in order to rule out clonality. Detecting clonality allows for distinguishing clonal eosinophilias that are associated with hematological malignancies from reactive eosinophilias that are associated with non-hematological disorders. 

The detection of clonality in eosinophilia, such as *STAT5B* mutations or tyrosine kinase fusion genes, could enable the use of targeted therapies, leading to an improvement in the quality of life and survival of patients.

## Figures and Tables

**Table 1:** Approach to eosinophilia.

Eosinophilia Categories			Supportive Features
Familial (hereditary) eosinophilia (with/without immunodeficiency)			Age, family history, and genetic study
Secondary (reactive) eosinophilia	Non-hematological	- Parasitosis - Infections - Allergic diseases - Drugs - Chronic inflammatory processes - Solid tumors	Clinical history and work-up focused on secondary causes
	Hematological	Lymphoid diseases	Hematologic work-up *
Primary (clonal, neoplastic) eosinophilia	Stem cell and myeloid neoplasms		Hematologic work-up *
Eosinophilia of unknown significance			Exclusion of previous disorders or conditions

* Hematologic work-up: peripheral blood and bone marrow examination with cytology, flow cytometry, histopathology, cytogenetics/fluorescence in situ hybridization, polymerase chain reaction, and other molecular techniques.

**Table 2:** Hematological neoplasms associated with eosinophilia.

Hematological neoplasms associated with reactive or secondary eosinophilia * Hodgkin lymphoma Mature T-cell neoplasms Lymphocytic variant of hypereosinophilic syndrome B-cell lymphoblastic leukemias/lymphomas T-lymphoblastic leukemias/lymphomas
Hematological neoplasm associated with neoplastic or primary eosinophilia ** Myeloid/lymphoid neoplasms with eosinophilia and defining gene rearrangements: 1.1. Myeloid/lymphoid neoplasms with *PDGFRA* rearrangement 1.2. Myeloid/lymphoid neoplasms with *PDGFRB* rearrangement 1.3. Myeloid/lymphoid neoplasms with *FGFR1* rearrangement 1.4. Myeloid/lymphoid neoplasms with *JAK2* rearrangement 1.5. Myeloid/lymphoid neoplasms with *FLT3* rearrangement 1.6. Myeloid/lymphoid neoplasms with *ETV6*::*ABL1* rearrangement 1.7. Myeloid/lymphoid neoplasms with other tyrosine kinase gene fusions Core binding factor acute myeloid leukemia 2.1. Acute myeloid leukemia with *CBFB*::*MYH11* fusion 2.2. Acute myeloid leukemia with *RUNX1*::*RUNX1T1* Mastocytosis Myeloproliferative neoplasms 4.1. Chronic myeloid leukemia 4.2. Chronic eosinophilic leukemia Myelodysplastic/Myeloproliferative neoplasms Myelodysplastic neoplasms

* In these disorders, eosinophilia is usually reactive to the release of eosinopoietic cytokines (polyclonal or benign). ** In these disorders, eosinophils are usually neoplastic (belonging to the malignant clone).

## References

[1] Ehrlich P. (1879). Über die spezifischen Granulationen des Blutes. Arch. Anat. Physiol..

[2] Kay A.B. (2015). The early history of the eosinophil. Clin. Exp. Allergy.

[3] Woessner Casas S., Florensa Brich L. (2006). La Citología Óptica en el Diagnóstico Hematológico.

[4] Johnston L.K., Bryce P.J. (2017). Understanding Interleukin 33 and Its Roles in Eosinophil Development. Front. Med..

[5] Varricchi G., Galdiero M.R., Loffredo S., Lucarini V., Marone G., Mattei F., Marone G., Schiavoni G. (2017). Eosinophils: The unsung heroes in cancer?. Oncoimmunology.

[6] DiScipio R.G., Schraufstatter I.U. (2007). The role of the complement anaphylatoxins in the recruitment of eosinophils. Int. Immunopharmacol..

[7] Melo R.C.N., Weller P.F. (2018). Contemporary understanding of the secretory granules in human eosinophils. J. Leukoc. Biol..

[8] Acharya K.R., Ackerman S.J. (2014). Eosinophil granule proteins: Form and function. J. Biol. Chem..

[9] Kanda A., Yasutaka Y., Van Bui D., Suzuki K., Sawada S., Kobayashi Y., Asako M., Iwai H. (2020). Multiple Biological Aspects of Eosinophils in Host Defense, Eosinophil-Associated Diseases, Immunoregulation, and Homeostasis: Is Their Role Beneficial, Detrimental, Regulator, or Bystander?. Biol. Pharm. Bull..

[10] Goasguen J.E., Bennett J.M., Bain B.J., Brunning R., Zini G., Vallespi M.T., Tomonaga M., Locher C., International Working Group on Morphology of MDS (2020). The role of eosinophil morphology in distinguishing between reactive eosinophilia and eosinophilia as a feature of a myeloid neoplasm. Br. J. Haematol..

[11] Zederbauer M., Furtmüller P.G., Brogioni S., Jakopitsch C., Smulevich G., Obinger C. (2007). Heme to protein linkages in mammalian peroxidases: Impact on spectroscopic, redox and catalytic properties. Nat. Prod. Rep..

[12] Orfao A., Matarraz S., Pérez-Andrés M., Almeida J., Teodosio C., Berkowska M.A., van Dongen J.J.M., EuroFlow (2019). Immunophenotypic dissection of normal hematopoiesis. J. Immunol. Methods.

[13] Bain B.J. (1996). Eosinophilic leukaemias and the idiopathic hypereosinophilic syndrome. Br. J. Haematol..

[14] Valent P., Klion A.D., Horny H.P., Roufosse F., Gotlib J., Weller P.F., Hellmann A., Metzgeroth G., Leiferman K.M., Arock M. (2012). Contemporary consensus proposal on criteria and classification of eosinophilic disorders and related syndromes. J. Allergy Clin. Immunol..

[15] Valent P., Klion A.D., Roufosse F., Simon D., Metzgeroth G., Leiferman K.M., Schwaab J., Butterfield J.H., Sperr W.R., Sotlar K. (2023). Proposed refined diagnostic criteria and classification of eosinophil disorders and related syndromes. Allergy.

[16] Valent P., Degenfeld-Schonburg L., Sadovnik I., Horny H.P., Arock M., Simon H.U., Reiter A., Bochner B.S. (2021). Eosinophils and eosinophil-associated disorders: Immunological, clinical, and molecular complexity. Semin. Immunopathol..

[17] Delgado-Serrano J., Morales-Camacho R.M., Caballero-Velázquez T., García-Canale S., Vargas M.T., Prats-Martín C. (2020). Eosinophils engulfing platelets and with ring-shaped nuclei in nivolumab-associated eosinophilia. Br. J. Haematol..

[18] Morales-Camacho R.M., Prats-Martín C. (2019). Eosinophils with ring-shaped nuclei in a patient treated with adalimumab. Blood.

[19] Klion A.D. (2022). Approach to the patient with suspected hypereosinophilic syndrome. Hematol. Am. Soc. Hematol. Educ. Program..

[20] Khoury P., Akuthota P., Kwon N., Steinfeld J., Roufosse F. (2023). HES and EGPA: Two Sides of the Same Coin. Mayo Clin. Proc..

[21] Guenzel A.J., Smadbeck J.B., Golden C.L., Williamson C.M., Benevides Demasi J.C., Vasmatzis G., Pearce K.E., Olteanu H., Xu X., Hoppman N.L. (2021). Clinical utility of next generation sequencing to detect IGH/IL3 rearrangements [t(5;14)(q31.1;q32.1)] in B-lymphoblastic leukemia/lymphoma. Ann. Diagn. Pathol..

[22] Romagnoli S., Bartalucci N., Gesullo F., Balliu M., Bonifacio S., Fernandez A.G.L., Mannelli F., Bolognini D., Pelo E., Mecucci C. (2021). Nanopore sequencing for the screening of myeloid and lymphoid neoplasms with eosinophilia and rearrangement of PDGFRα, PDGFRβ, FGFR1 or PCM1-JAK2. Biomark. Res..

[23] Podvin B., Roynard P., Boudry A., Guermouche H., Daudignon A., Terriou L., Bouabdelli W., Salameh M., Grardel N., Duployez N. (2022). Whole-genome optical mapping to elucidate myeloid/lymphoid neoplasms with eosinophilia and tyrosine kinase gene fusions. Leuk. Res..

[24] Shomali W., Damnernsawad A., Theparee T., Sampson D., Morrow Q., Yang F., Fernandez-Pol S., Press R., Zehnder J., Tyner J.W. (2021). A novel activating JAK1 mutation in chronic eosinophilic leukemia. Blood Adv..

[25] Cross N.C.P., Hoade Y., Tapper W.J., Carreno-Tarragona G., Fanelli T., Jawhar M., Naumann N., Pieniak I., Lübke J., Ali S. (2019). Recurrent activating STAT5B N642H mutation in myeloid neoplasms with eosinophilia. Leukemia.

[26] Khoury J.D., Solary E., Abla O., Akkari Y., Alaggio R., Apperley J.F., Bejar R., Berti E., Busque L., Chan J.K.C. (2022). The 5th edition of the World Health Organization Classification of Haematolymphoid Tumours: Myeloid and Histiocytic/Dendritic Neoplasms. Leukemia..

[27] Yin C.C., Tam W., Walker S.M., Kaur A., Ouseph M.M., Xie W., Weinberg O.K., Li P., Zuo Z., Routbort M.J. (2023). STAT5B mutations in myeloid neoplasms differ by disease subtypes but characterize a subset of chronic myeloid neoplasms with eosinophilia and/or basophilia. Haematologica.

[28] Umrau K., Naganuma K., Gao Q., Dogan A., Kizaki M., Roshal M., Liu Y., Yabe M. (2023). Activating STAT5B mutations can cause both primary hypereosinophilia and lymphocyte-variant hypereosinophilia. Leuk. Lymphoma.

[29] Sreedharanunni S., Jamwal M., Balakrishnan A., Aravindan A.V., Sharma R., Singh N., Rajpal S., Singla S., Khadwal A.R., Ahluwalia J. (2022). Chronic eosinophilic leukemia with recurrent STAT5B N642H mutation-An entity with features of myelodysplastic syndrome/myeloproliferative neoplasm overlap. Leuk. Res..

[30] Ding F., Wu C., Li Y., Mukherjee S., Ghosh S., Arrossi A.V., Krishnan S. (2021). A case of hypereosinophilic syndrome with STAT5b N642H mutation. Oxf. Med. Case Rep..

[31] Ma C.A., Xi L., Cauff B., DeZure A., Freeman A.F., Hambleton S., Kleiner G., Leahy T.R., O’Sullivan M., Makiya M. (2017). Somatic STAT5b gain-of-function mutations in early onset nonclonal eosinophilia, urticaria, dermatitis, and diarrhea. Blood.

[32] Roufosse F., Garaud S., de Leval L. (2012). Lymphoproliferative disorders associated with hypereosinophilia. Semin. Hematol..

[33] Samoszuk M., Nansen L. (1990). Detection of interleukin-5 messenger RNA in Reed-Sternberg cells of Hodgkin’s disease with eosinophilia. Blood.

[34] Muñoz-García N., Lima M., Villamor N., Morán-Plata F.J., Barrena S., Mateos S., Caldas C., Balanzategui A., Alcoceba M., Domínguez A. (2021). Anti-TRBC1 Antibody-Based Flow Cytometric Detection of T-Cell Clonality: Standardization of Sample Preparation and Diagnostic Implementation. Cancers.

[35] Tancrède-Bohin E., Ionescu M.A., de La Salmonière P., Dupuy A., Rivet J., Rybojad M., Dubertret L., Bachelez H., Lebbé C., Morel P. (2004). Prognostic value of blood eosinophilia in primary cutaneous T-cell lymphomas. Arch. Dermatol..

[36] Pulitzer M.P., Horna P., Almeida J. (2021). Sézary syndrome and mycosis fungoides: An overview, including the role of immunophenotyping. Cytometry B Clin. Cytom..

[37] Pu Q., Qiao J., Liu Y., Cao X., Tan R., Yan D., Wang X., Li J., Yue B. (2022). Differential diagnosis and identification of prognostic markers for peripheral T-cell lymphoma subtypes based on flow cytometry immunophenotype profiles. Front. Immunol..

[38] Tamaki T., Karube K., Sakihama S., Tsuruta Y., Awazawa R., Hayashi M., Nakada N., Matsumoto H., Yagi N., Ohshiro K. (2023). A Comprehensive Study of the Immunophenotype and its Clinicopathologic Significance in Adult T-Cell Leukemia/Lymphoma. Mod. Pathol..

[39] Shi Y., Wang C. (2022). What we have learned about lymphocytic variant hypereosinophilic syndrome: A systematic literature review. Clin. Immunol..

[40] Arber D.A., Orazi A., Hasserjian R.P., Borowitz M.J., Calvo K.R., Kvasnicka H.M., Wang S.A., Bagg A., Barbui T., Branford S. (2022). International Consensus Classification of Myeloid Neoplasms and Acute Leukemias: Integrating morphologic, clinical, and genomic data. Blood.

[41] Fournier B., Balducci E., Duployez N., Clappier E., Cuccuini W., Arfeuille C., Caye-Eude A., Delabesse E., Bottollier-Lemallaz C.E., Nebral K. (2019). B-ALL with t(5;14)(q31;q32); IGH-IL3 Rearrangement and Eosinophilia: A Comprehensive Analysis of a Peculiar IGH-Rearranged B-ALL. Front. Oncol..

[42] McClure B.J., Heatley S.L., Rehn J., Breen J., Sutton R., Hughes T.P., Suppiah R., Revesz T., Osborn M., White D. (2020). High-risk B-cell acute lymphoblastic leukaemia presenting with hypereosinophilia and acquiring a novel PAX5 fusion on relapse. Br. J. Haematol..

[43] Kwon A., Fuda F., Gagan J., John S., Aquino V., Chen W. (2023). Rare circulating lymphoblasts with striking eosinophilia: A rare case of B-lymphoblastic leukemia with PAX5::ZCCHC7. Am. J. Hematol..

[44] Ferruzzi V., Santi E., Gurdo G., Arcioni F., Caniglia M., Esposito S. (2018). Acute Lymphoblastic Leukemia with Hypereosinophilia in a Child: Case Report and Literature Review. Int. J. Environ. Res. Public Health.

[45] Pierini V., Bardelli V., Giglio F., Arniani S., Matteucci C., Pellanera F., Quintini M., Crescenzi B., Bruno A., Sabattini E. (2022). A Novel t(5;7)(q31;q21)/CDK6::IL3 in Immature T-cell Acute Lymphoblastic Leukemia With IL3 Expression and Eosinophilia. Hemasphere.

[46] Swerdlow S.H., Campo E., Harris N.L., Jaffe E.S., Pileri S.A., Stein H., Thiele J. (2017). WHO Classification of Tumours of Haematopoietic and Lymphoid Tissues.

[47] Kim A.S., Pozdnyakova O. (2022). SOHO State of the Art Updates and Next Questions|Myeloid/Lymphoid Neoplasms with Eosinophilia and Gene Rearrangements: Diagnostic Pearls and Pitfalls. Clin. Lymphoma Myeloma Leuk..

[48] Pozdnyakova O., Orazi A., Kelemen K., King R., Reichard K.K., Craig F.E., Quintanilla-Martinez L., Rimsza L., George T.I., Horny H.P. (2021). Myeloid/Lymphoid Neoplasms Associated with Eosinophilia and Rearrangements of PDGFRA, PDGFRB, or FGFR1 or With PCM1-JAK2. Am. J. Clin. Pathol..

[49] Metzgeroth G., Steiner L., Naumann N., Lübke J., Kreil S., Fabarius A., Haferlach C., Haferlach T., Hofmann W.K., Reiter A. (2023). Myeloid/lymphoid neoplasms with eosinophilia and tyrosine kinase gene fusions: Reevaluation of the defining characteristics in a registry-based cohort. Leukemia.

[50] Saft L., Kvasnicka H.M., Boudova L., Gianelli U., Lazzi S., Rozman M. (2023). Myeloid/lymphoid neoplasms with eosinophilia and tyrosine kinase fusion genes: A workshop report with focus on novel entities and a literature review including paediatric cases. Histopathology.

[51] Shomali W., Gotlib J. (2022). World Health Organization-defined eosinophilic disorders: 2022 update on diagnosis, risk stratification, and management. Am. J. Hematol..

[52] Rohmer J., Couteau-Chardon A., Trichereau J., Panel K., Gesquiere C., Ben Abdelali R., Bidet A., Bladé J.S., Cayuela J.M., Cony-Makhoul P. (2020). Epidemiology, clinical picture and long-term outcomes of FIP1L1-PDGFRA-positive myeloid neoplasm with eosinophilia: Data from 151 patients. Am. J. Hematol..

[53] Score J., Curtis C., Waghorn K., Stalder M., Jotterand M., Grand F.H., Cross N.C. (2006). Identification of a novel imatinib responsive KIF5B-PDGFRA fusion gene following screening for PDGFRA overexpression in patients with hypereosinophilia. Leukemia.

[54] Walz C., Curtis C., Schnittger S., Schultheis B., Metzgeroth G., Schoch C., Lengfelder E., Erben P., Müller M.C., Haferlach T. (2006). Transient response to imatinib in a chronic eosinophilic leukemia associated with ins(9;4)(q33;q12q25) and a CDK5RAP2-PDGFRA fusion gene. Genes Chromosomes Cancer.

[55] Curtis C.E., Grand F.H., Musto P., Clark A., Murphy J., Perla G., Minervini M.M., Stewart J., Reiter A., Cross N.C. (2007). Two novel imatinib-responsive PDGFRA fusion genes in chronic eosinophilic leukaemia. Br. J. Haematol..

[56] Baxter E.J., Hochhaus A., Bolufer P., Reiter A., Fernandez J.M., Senent L., Cervera J., Moscardo F., Sanz M.A., Cross N.C. (2002). The t(4;22)(q12;q11) in atypical chronic myeloid leukaemia fuses BCR to PDGFRA. Hum. Mol. Genet..

[57] Chalmers Z.R., Ali S.M., Ohgami R.S., Campregher P.V., Frampton G.M., Yelensky R., Elvin J.A., Palma N.A., Erlich R., Vergilio J.A. (2015). Comprehensive genomic profiling identifies a novel TNKS2-PDGFRA fusion that defines a myeloid neoplasm with eosinophilia that responded dramatically to imatinib therapy. Blood Cancer J..

[58] Sugimoto Y., Sada A., Shimokariya Y., Monma F., Ohishi K., Masuya M., Nobori T., Matsui T., Katayama N. (2015). A novel FOXP1-PDGFRA fusion gene in myeloproliferative neoplasm with eosinophilia. Cancer Genet..

[59] Baer C., Muehlbacher V., Kern W., Haferlach C., Haferlach T. (2018). Molecular genetic characterization of myeloid/lymphoid neoplasms associated with eosinophilia and rearrangement of PDGFRA, PDGFRB, FGFR1 or PCM1-JAK2. Haematologica.

[60] Jain N., Khoury J.D., Pemmaraju N., Kollipara P., Kantarjian H., Verstovsek S. (2013). Imatinib therapy in a patient with suspected chronic neutrophilic leukemia and FIP1L1-PDGFRA rearrangement. Blood.

[61] Huang Q., Snyder D.S., Chu P., Gaal K.K., Chang K.L., Weiss L.M. (2011). PDGFRA rearrangement leading to hyper-eosinophilia, T-lymphoblastic lymphoma, myeloproliferative neoplasm and precursor B-cell acute lymphoblastic leukemia. Leukemia.

[62] Trempat P., Villalva C., Laurent G., Armstrong F., Delsol G., Dastugue N., Brousset P. (2003). Chronic myeloproliferative disorders with rearrangement of the platelet-derived growth factor alpha receptor: A new clinical target for STI571/Glivec. Oncogene.

[63] Akiely R., Almasri F., Almasri N., Abu-Ghosh A. (2022). Case Report: Pediatric myeloid/lymphoid neoplasm with eosinophilia and PDGFRA rearrangement: The first case presenting as B-lymphoblastic lymphoma. Front. Pediatr..

[64] Roberts K.G., Gu Z., Payne-Turner D., McCastlain K., Harvey R.C., Chen I.M., Pei D., Iacobucci I., Valentine M., Pounds S.B. (2017). High Frequency and Poor Outcome of Philadelphia Chromosome-Like Acute Lymphoblastic Leukemia in Adults. J. Clin. Oncol..

[65] Krigstein M., Menzies A., Fay K., Lukeis R., Cheung K., Parker A. (2023). FIP1L1::PDGFRA fusion driving three synchronous haematological malignancies. Pathology.

[66] Bellani V., Croci G.A., Bucelli C., Maronese C.A., Alberti S., Iurlo A., Cattaneo D. (2023). Lymphomatoid papulosis associated with myeloid neoplasm with eosinophilia and FIP1L1::PDGFRA rearrangement: Successful imatinib treatment in two cases. J. Dermatol..

[67] Baccarani M., Cilloni D., Rondoni M., Ottaviani E., Messa F., Merante S., Tiribelli M., Buccisano F., Testoni N., Gottardi E. (2007). The efficacy of imatinib mesylate in patients with FIP1L1-PDGFRalpha-positive hypereosinophilic syndrome. Results of a multicenter prospective study. Haematologica.

[68] Pardanani A., D’Souza A., Knudson R.A., Hanson C.A., Ketterling R.P., Tefferi A. (2012). Long-term follow-up of FIP1L1-PDGFRA-mutated patients with eosinophilia: Survival and clinical outcome. Leukemia.

[69] Jawhar M., Naumann N., Schwaab J., Baurmann H., Casper J., Dang T.A., Dietze L., Döhner K., Hänel A., Lathan B. (2017). Imatinib in myeloid/lymphoid neoplasms with eosinophilia and rearrangement of PDGFRB in chronic or blast phase. Ann. Hematol..

[70] Cheah C.Y., Burbury K., Apperley J.F., Huguet F., Pitini V., Gardembas M., Ross D.M., Forrest D., Genet P., Rousselot P. (2014). Patients with myeloid malignancies bearing PDGFRB fusion genes achieve durable long-term remissions with imatinib. Blood.

[71] Di Giacomo D., Quintini M., Pierini V., Pellanera F., La Starza R., Gorello P., Matteucci C., Crescenzi B., Fiumara P.F., Veltroni M. (2022). Genomic and clinical findings in myeloid neoplasms with PDGFRB rearrangement. Ann. Hematol..

[72] Panagopoulos I., Brunetti M., Stoltenberg M., Strandabø R.A.U., Staurseth J., Andersen K., Kostolomov I., Hveem T.S., Lorenz S., Nystad T.A. (2019). Novel GTF2I-PDGFRB and IKZF1-TYW1 fusions in pediatric leukemia with normal karyotype. Exp. Hematol. Oncol..

[73] Bain B.J., Fletcher S.H. (2007). Chronic eosinophilic leukemias and the myeloproliferative variant of the hypereosinophilic syndrome. Immunol. Allergy Clin. N. Am..

[74] Schwab C., Ryan S.L., Chilton L., Elliott A., Murray J., Richardson S., Wragg C., Moppett J., Cummins M., Tunstall O. (2016). EBF1-PDGFRB fusion in pediatric B-cell precursor acute lymphoblastic leukemia (BCP-ALL): Genetic profile and clinical implications. Blood.

[75] Heilmann A.M., Schrock A.B., He J., Nahas M., Curran K., Shukla N., Cramer S., Draper L., Verma A., Erlich R. (2017). Novel PDGFRB Fusions in Childhood B- and T-Acute Lymphoblastic Leukemia. Leukemia.

[76] Maccaferri M., Pierini V., Di Giacomo D., Zucchini P., Forghieri F., Bonacorsi G., Paolini A., Quadrelli C., Giacobbi F., Fontana F. (2017). The Importance of Cytogenetic and Molecular Analyses in Eosinophilia-Associated Myeloproliferative Neoplasms: An Unusual Case with Normal Karyotype and TNIP1-PDGFRB Rearrangement and Overview of PDGFRB Partner Genes. Leuk. Lymphoma.

[77] Reiter A., Gotlib J. (2017). Myeloid Neoplasms with Eosinophilia. Blood.

[78] Darbyshire P.J., Shortland D., Swansbury G.J., Sadler J., Lawler S.D., Chessells J.M. (1987). A Myeloproliferative Disease in Two Infants Associated with Eosinophilia and Chromosome t(1;5) Translocation. Br. J. Haematol..

[79] Wilkinson K., Velloso E.R., Lopes L.F., Lee C., Aster J.C., Shipp M.A., Aguiar R.C. (2003). Cloning of the t(1;5)(q23;q33) in a Myeloproliferative Disorder Associated with Eosinophilia: Involvement of PDGFRB and Response to Imatinib. Blood.

[80] Li Z., Yang R., Zhao J., Yuan R., Lu Q., Li Q., Tse W. (2011). Molecular Diagnosis and Targeted Therapy of a Pediatric Chronic Eosinophilic Leukemia Patient Carrying TPM3-PDGFRB Fusion. Pediatr. Blood Cancer.

[81] Abraham S., Salama M., Hancock J., Jacobsen J., Fluchel M. (2012). Congenital and Childhood Myeloproliferative Disorders with Eosinophilia Responsive to Imatinib. Pediatr. Blood Cancer.

[82] Berking A.C., Flaadt T., Behrens Y.L., Yoshimi A., Leipold A., Holzer U., Lang P., Quintanilla-Martinez L., Schlegelberger B., Reiter A. (2023). Rare and Potentially Fatal—Cytogenetically Cryptic TNIP1::PDGFRB and PCM1::FGFR1 Fusion Leading to Myeloid/Lymphoid Neoplasms with Eosinophilia in Children. Cancer Genet..

[83] Wang S.C., Yang W.Y. (2021). Myeloid Neoplasm with Eosinophilia and Rearrangement of Platelet-Derived Growth Factor Receptor Beta Gene in Children: Two Case Reports. World J. Clin. Cases.

[84] McKeague S.J., O’Rourke K., Fanning S., Joy C., Throp D., Adams R., Harvey Y., Keng T.B. (2023). Acute Leukemia with Cytogenetically Cryptic FGFR1 Rearrangement and Lineage Switch during Therapy: A Case Report and Literature Review. Am. J. Clin. Pathol..

[85] Strati P., Tang G., Duose D.Y., Mallampati S., Luthra R., Patel K.P., Hussaini M., Mirza A.S., Komrokji R.S., Oh S. (2018). Myeloid/Lymphoid Neoplasms with FGFR1 Rearrangement. Leuk. Lymphoma.

[86] Hernández-Boluda J.C., Pereira A., Zinger N., Gras L., Martino R., Nikolousis E., Finke J., Chinea A., Rambaldi A., Robin M. (2022). Allogeneic Hematopoietic Cell Transplantation in Patients with Myeloid/Lymphoid Neoplasm with FGFR1-Rearrangement: A Study of the Chronic Malignancies Working Party of EBMT. Bone Marrow Transplant..

[87] Parasuraman S., Teschemaker A., Kish J.K., Savill K.M., Colucci P. (2022). Myeloid/Lymphoid Neoplasms (MLNs) with Fibroblast Growth Factor Receptor 1 (FGFR1) Rearrangement (MLNFGFR1): A US Real-World Retrospective Cohort Study. Blood.

[88] Wakim J.J., Tirado C.A., Chen W., Collins R. (2011). t(8;22)/BCR-FGFR1 Myeloproliferative Disorder Presenting as B-Acute Lymphoblastic Leukemia: Report of a Case Treated with Sorafenib and Review of the Literature. Leuk. Res..

[89] Haslam K., Langabeer S.E., Kelly J., Coen N., O’Connell N.M., Conneally E. (2012). Allogeneic Hematopoietic Stem Cell Transplantation for a BCR-FGFR1 Myeloproliferative Neoplasm Presenting as Acute Lymphoblastic Leukemia. Case Rep. Hematol..

[90] Baldazzi C., Iacobucci I., Luatti S., Ottaviani E., Marzocchi G., Paolini S., Stacchini M., Papayannidis C., Gamberini C., Martinelli G. (2010). B-Cell Acute Lymphoblastic Leukemia as Evolution of a 8p11 Myeloproliferative Syndrome with t(8;22)(p11;q11) and BCR-FGFR1 Fusion Gene. Leuk. Res..

[91] Macdonald D., Aguiar R.C., Mason P.J., Goldman J.M., Cross N.C. (1995). A New Myeloproliferative Disorder Associated with Chromosomal Translocations Involving 8p11: A Review. Leukemia.

[92] Macdonald D., Reiter A., Cross N.C. (2002). The 8p11 Myeloproliferative Syndrome: A Distinct Clinical Entity Caused by Constitutive Activation of FGFR1. Acta Haematol..

[93] Nakayama H., Inamitsu T., Ohga S., Kai T., Suda M., Matsuzaki A., Ueda K. (1996). Chronic Myelomonocytic Leukaemia with t(8;9)(p11;q34) in Childhood: An Example of the 8p11 Myeloproliferative Disorder?. Br. J. Haematol..

[94] van den Berg H., Kroes W., van der Schoot C.E., Dee R., Pals S.T., Bouts T.H., Slater R.M. (1996). A Young Child with Acquired t(8;9)(p11;q34): Additional Proof That 8p11 Is Involved in Mixed Myeloid/T Lymphoid Malignancies. Leukemia.

[95] Wong W.S., Cheng K.C., Lau K.M., Chan N.P., Shing M.M., Cheng S.H., Chik K.W., Li C.K., Ng M.H. (2007). Clonal Evolution of 8p11 Stem Cell Syndrome in a 14-Year-Old Chinese Boy: A Review of Literature of t(8;13) Associated Myeloproliferative Diseases. Leuk. Res..

[96] Zhang W.W., Habeebu S., Sheehan A.M., Naeem R., Hernandez V.S., Dreyer Z.E., López-Terrada D. (2009). Molecular Monitoring of 8p11 Myeloproliferative Syndrome in an Infant. J. Pediatr. Hematol. Oncol..

[97] Yan Y., Qu S., Liu J., Li C., Yan X., Xu Z., Qin T., Jia Y., Pan L., Gao Q. (2023). Olverembatinib for Myeloid/Lymphoid Neoplasm Associated with Eosinophilia and FGFR1 Rearrangement. Leuk. Lymphoma.

[98] Ren M., Qin H., Ren R., Cowell J.K. (2013). Ponatinib Suppresses the Development of Myeloid and Lymphoid Malignancies Associated with FGFR1 Abnormalities. Leukemia.

[99] Chen J., DeAngelo D.J., Kutok J.L., Williams I.R., Lee B.H., Wadleigh M., Duclos N., Cohen S., Adelsperger J., Okabe R. (2004). PKC412 Inhibits the Zinc Finger 198-Fibroblast Growth Factor Receptor 1 Fusion Tyrosine Kinase and Is Active in Treatment of Stem Cell Myeloproliferative Disorder. Proc. Natl. Acad. Sci. USA.

[100] Kasbekar M., Nardi V., Dal Cin P., Brunner A.M., Burke M., Chen Y.B., Connolly C., Fathi A.T., Foster J., Macrae M. (2020). Targeted FGFR Inhibition Results in a Durable Remission in an FGFR1-Driven Myeloid Neoplasm with Eosinophilia. Blood Adv..

[101] Verstovsek S., Vannucchi A.M., Rambaldi A., Gotlib M.J.R., Mead A.J., Hochhaus A., Kiladjian J.-J., Boluda J.C.H., Asatiani E., Lihou B.C. (2018). Interim Results from Fight-203, a Phase 2, Open-Label, Multicenter Study Evaluating the Efficacy and Safety of Pemigatinib (INCB054828) in Patients with Myeloid/Lymphoid Neoplasms with Rearrangement of Fibroblast Growth Factor Receptor 1 (FGFR1). Blood.

[102] Tzankov A., Reichard K.K., Hasserjian R.P., Arber D.A., Orazi A., Wang S.A. (2023). Updates on Eosinophilic Disorders. Virchows Arch..

[103] Kaplan H.G., Jin R., Bifulco C.B., Scanlan J.M., Corwin D.R. (2022). PCM1-JAK2 Fusion Tyrosine Kinase Gene-Related Neoplasia: A Systematic Review of the Clinical Literature. Oncologist.

[104] Rumi E., Milosevic J.D., Casetti I., Dambruoso I., Pietra D., Boveri E., Boni M., Bernasconi P., Passamonti F., Kralovics R. (2013). Efficacy of Ruxolitinib in Chronic Eosinophilic Leukemia Associated with a PCM1-JAK2 Fusion Gene. J. Clin. Oncol..

[105] Lierman E., Selleslag D., Smits S., Billiet J., Vandenberghe P. (2012). Ruxolitinib Inhibits Transforming JAK2 Fusion Proteins In Vitro and Induces Complete Cytogenetic Remission in t(8;9)(p22;p24)/PCM1-JAK2-Positive Chronic Eosinophilic Leukemia. Blood.

[106] Tang G., Tam W., Short N.J., Bose P., Wu D., Hurwitz S.N., Bagg A., Rogers H.J., Hsi E.D., Quesada A.E. (2021). Myeloid/Lymphoid Neoplasms with FLT3 Rearrangement. Mod. Pathol..

[107] Venable E.R., Gagnon M.F., Pitel B.A., Palmer J.M., Peterson J.F., Baughn L.B., Hoppman N.L., Greipp P.T., Ketterling R.P., Patnaik M.S. (2023). A TRIP11::FLT3 Gene Fusion in a Patient with Myeloid/Lymphoid Neoplasm with Eosinophilia and Tyrosine Kinase Gene Fusions: A Case Report and Review of the Literature. Cold Spring Harb. Mol. Case Stud..

[108] Schoelinck J., Gervasoni J., Guillermin Y., Beillard E., Pissaloux D., Chassagne-Clement C. (2023). T Cell Phenotype and Lack of Eosinophilia Are Not Uncommon in Extramedullary Myeloid/Lymphoid Neoplasms with ETV6::FLT3 Fusion: A Case Report and Review of the Literature. Virchows Arch..

[109] Spitzer B., Dela Cruz F.S., Ibanez Sanchez G.D., Zhang Y., Xiao W., Benayed R., Markova A., Rodriguez-Sanchez M.I., Bouvier N., Roshal M. (2021). ETV6-FLT3-Positive Myeloid/Lymphoid Neoplasm with Eosinophilia Presenting in an Infant: An Entity Distinct from JMML. Blood Adv..

[110] Munthe-Kaas M.C., Forthun R.B., Brendehaug A., Eek A.K., Høysæter T., Osnes L.T.N., Prescott T., Spetalen S., Hovland R. (2021). Partial Response to Sorafenib in a Child with a Myeloid/Lymphoid Neoplasm, Eosinophilia, and a ZMYM2-FLT3 Fusion. J. Pediatr. Hematol. Oncol..

[111] Falchi L., Mehrotra M., Newberry K.J., Lyle L.M., Lu G., Patel K.P., Luthra R., Popat U., Verstovsek S. (2014). ETV6-FLT3 Fusion Gene-Positive, Eosinophilia-Associated Myeloproliferative Neoplasm Successfully Treated with Sorafenib and Allogeneic Stem Cell Transplant. Leukemia.

[112] Walz C., Erben P., Ritter M., Bloor A., Metzgeroth G., Telford N., Haferlach C., Haferlach T., Gesk S., Score J. (2011). Response of ETV6-FLT3-Positive Myeloid/Lymphoid Neoplasm with Eosinophilia to Inhibitors of FMS-Like Tyrosine Kinase 3. Blood.

[113] Chonabayashi K., Hishizawa M., Matsui M., Kondo T., Ohno T., Ishikawa T., Takaori-Kondo A. (2014). Successful Allogeneic Stem Cell Transplantation with Long-Term Remission of ETV6/FLT3-Positive Myeloid/Lymphoid Neoplasm with Eosinophilia. Ann. Hematol..

[114] Tasian S.K., Loh M.L., Hunger S.P. (2017). Philadelphia Chromosome-Like Acute Lymphoblastic Leukemia. Blood.

[115] De Braekeleer E., Douet-Guilbert N., Rowe D., Bown N., Morel F., Berthou C., Férec C., De Braekeleer M. (2011). ABL1 Fusion Genes in Hematological Malignancies: A Review. Eur. J. Haematol..

[116] Zaliova M., Moorman A.V., Cazzaniga G., Stanulla M., Harvey R.C., Roberts K.G., Heatley S.L., Loh M.L., Konopleva M., Chen I.M. (2016). Characterization of Leukemias with ETV6-ABL1 Fusion. Haematologica.

[117] Schwaab J., Naumann N., Luebke J., Jawhar M., Somervaille T.C.P., Williams M.S., Frewin R., Jost P.J., Lichtenegger F.S., La Rosée P. (2020). Response to Tyrosine Kinase Inhibitors in Myeloid Neoplasms Associated with PCM1-JAK2, BCR-JAK2, and ETV6-ABL1 Fusion Genes. Am. J. Hematol..

[118] Carll T., Patel A., Derman B., Hyjek E., Lager A., Wanjari P., Segal J., Odenike O., Fidai S., Arber D. (2020). Diagnosis and Treatment of Mixed Phenotype (T-Myeloid/Lymphoid) Acute Leukemia with Novel ETV6-FGFR2 Rearrangement. Blood Adv..

[119] Telford N., Alexander S., McGinn O.J., Williams M., Wood K.M., Bloor A., Saha V. (2016). Myeloproliferative Neoplasm with Eosinophilia and T-Lymphoblastic Lymphoma with ETV6-LYN Gene Fusion. Blood Cancer J..

[120] Kralik J.M., Kranewitter W., Boesmueller H., Marschon R., Tschurtschenthaler G., Rumpold H., Wiesinger K., Erdel M., Petzer A.L., Webersinke G. (2011). Characterization of a Newly Identified ETV6-NTRK3 Fusion Transcript in Acute Myeloid Leukemia. Diagn. Pathol..

[121] Maesako Y., Izumi K., Okamori S., Takeoka K., Kishimori C., Okumura A., Honjo G., Akasaka T., Ohno H. (2014). Inv(2)(p23q13)/RAN-Binding Protein 2 (RANBP2)-ALK Fusion Gene in Myeloid Leukemia That Developed in an Elderly Woman. Int. J. Hematol..

[122] Ballerini P., Struski S., Cresson C., Prade N., Toujani S., Deswarte C., Dobbelstein S., Petit A., Lapillonne H., Gautier E.F. (2012). RET Fusion Genes Are Associated with Chronic Myelomonocytic Leukemia and Enhance Monocytic Differentiation. Leukemia.

[123] Roberts K.G., Janke L.J., Zhao Y., Seth A., Ma J., Finkelstein D., Smith S., Ebata K., Tuch B.B., Hunger S.P. (2018). ETV6-NTRK3 Induces Aggressive Acute Lymphoblastic Leukemia Highly Sensitive to Selective TRK Inhibition. Blood.

[124] Maesako Y., Okumura A., Takeoka K., Kishimori C., Izumi K., Kamoda Y., Iioka F., Akasaka T., Ohno H. (2014). Reduction of Leukemia Cell Burden and Restoration of Normal Hematopoiesis at 3 Months of Crizotinib Treatment in RAN-Binding Protein 2 (RANBP2)-Anaplastic Lymphoma Kinase (ALK) Acute Myeloid Leukemia. Leukemia.

[125] Naymagon L., Marcellino B., Mascarenhas J. (2019). Eosinophilia in Acute Myeloid Leukemia: Overlooked and Underexamined. Blood Rev..

[126] Larson R.A., Williams S.F., Le Beau M.M., Bitter M.A., Vardiman J.W., Rowley J.D. (1986). Acute Myelomonocytic Leukemia with Abnormal Eosinophils and Inv(16) or t(16;16) Has a Favorable Prognosis. Blood.

[127] Ouyang J., Goswami M., Peng J., Zuo Z., Daver N., Borthakur G., Tang G., Medeiros L.J., Jorgensen J.L., Ravandi F. (2016). Comparison of Multiparameter Flow Cytometry Immunophenotypic Analysis and Quantitative RT-PCR for the Detection of Minimal Residual Disease of Core Binding Factor Acute Myeloid Leukemia. Am. J. Clin. Pathol..

[128] Raya J.M., Gómez-Hernando M., Guijarro F., Alonso E., Arnan M., Senent L., Vicente A.I., Borrego A., Gómez-Casares M.T., González-González C. (2021). PO-115: Leucemia Aguda Mieloide con t(8,21); RUNX1-RUNX1T1: Análisis Multicéntrico Retrospectivo de 165 Pacientes. Proceedings of the LXIII Congreso Nacional de la SEHH XXXVII Congreso Nacional de la SETH.

[129] Swirsky D.M., Li Y.S., Matthews J.G., Flemans R.J., Rees J.K., Hayhoe F.G. (1984). 8;21 Translocation in Acute Granulocytic Leukaemia: Cytological, Cytochemical and Clinical Features. Br. J. Haematol..

[130] Haferlach T., Bennett J.M., Löffler H., Gassmann W., Andersen J.W., Tuzuner N., Casslleth P.A., Fonatsch C., Schoch C., Schlegelberger B. (1996). Acute Myeloid Leukemia with Translocation (8;21). Cytomorphology, Dysplasia, and Prognostic Factors in 41 Cases. AML Cooperative Group and ECOG. Leuk. Lymphoma.

[131] Valent P., Akin C., Hartmann K., Alvarez-Twose I., Brockow K., Hermine O., Niedoszytko M., Schwaab J., Lyons J.J., Carter M.C. (2021). Updated Diagnostic Criteria and Classification of Mast Cell Disorders: A Consensus Proposal. Hemasphere.

[132] Kluin-Nelemans H.C., Reiter A., Illerhaus A., van Anrooij B., Hartmann K., Span L.F.R., Gorska A., Niedoszytko M., Lange M., Scaffidi L. (2020). Prognostic Impact of Eosinophils in Mastocytosis: Analysis of 2350 Patients Collected in the ECNM Registry. Leukemia.

[133] Kovalszki A., Weller P.F. (2014). Eosinophilia in Mast Cell Disease. Immunol. Allergy Clin. N. Am..

[134] Morales-Camacho R.M., Villanueva-Herraiz S., Prats-Martín C., Borrero J.J., Bernal R., Vargas M.T. (2018). Eosinophil Phagocytosis in Advanced Systemic Mastocytosis with Eosinophilia. Br. J. Haematol..

[135] Moshref Razavi H., Yu R. (2022). Myeloid Neoplasm with PDGFRB Rearrangement, Presenting as Systemic Mastocytosis-Chronic Eosinophilic Leukemia. Am. J. Hematol..

[136] Leguit R.J., Wang S.A., George T.I., Tzankov A., Orazi A. (2023). The International Consensus Classification of Mastocytosis and Related Entities. Virchows Arch..

[137] Morgado J.M., Perbellini O., Johnson R.C., Teodósio C., Matito A., Álvarez-Twose I., Bonadonna P., Zamò A., Jara-Acevedo M., Mayado A. (2013). CD30 Expression by Bone Marrow Mast Cells from Different Diagnostic Variants of Systemic Mastocytosis. Histopathology.

[138] Duckworth C.B., Zhang L., Li S. (2014). Systemic Mastocytosis with Associated Myeloproliferative Neoplasm with t(8;19)(p12;q13.1) and Abnormality of FGFR1: Report of a Unique Case. Int. J. Clin. Exp. Pathol..

[139] Langabeer S.E. (2021). The Eosinophilic Variant of Chronic Myeloid Leukemia. EXCLI J..

[140] Morsia E., Reichard K., Pardanani A., Tefferi A., Gangat N. (2020). WHO Defined Chronic Eosinophilic Leukemia, Not Otherwise Specified (CEL, NOS): A Contemporary Series from the Mayo Clinic. Am. J. Hematol..

[141] Matsushima T., Handa H., Yokohama A., Nagasaki J., Koiso H., Kin Y., Tanaka Y., Sakura T., Tsukamoto N., Karasawa M. (2003). Prevalence and Clinical Characteristics of Myelodysplastic Syndrome with Bone Marrow Eosinophilia or Basophilia. Blood.

[142] Patnaik M.M., Gangat N., Knudson R.A., Keefe J.G., Hanson C.A., Pardanani A., Ketterling R.P., Tefferi A. (2010). Chromosome 8p11.2 Translocations: Prevalence, FISH Analysis for FGFR1 and MYST3, and Clinicopathologic Correlates in a Consecutive Cohort of 13 Cases from a Single Institution. Am. J. Hematol..

[143] Tang G., Sydney Sir Philip J.K., Weinberg O., Tam W., Sadigh S., Lake J.I., Margolskee E.M., Rogers H.J., Miranda R.N., Bueso-Ramos C.C. (2019). Hematopoietic Neoplasms with 9p24/JAK2 Rearrangement: A Multicenter Study. Mod. Pathol..

